# Microglial *Rack1* Deficiency Alleviates Alzheimer's Disease Pathology through Enhancing IGF1‐Mediated Astrocytic Phagocytosis

**DOI:** 10.1002/advs.202515877

**Published:** 2025-10-30

**Authors:** Jingdan Zhang, Yanling He, Pin Yang, Hengchang Zhang, Yueyang Tong, Lingling Jiang, Zehan Li, Meichen Yan, Xiaoheng Li, Qinghu Yang, Jing Yang, Zengqiang Yuan, Jiyan Zhang, Jinbo Cheng

**Affiliations:** ^1^ Center on Translational Neuroscience, College of Life & Environmental Science Minzu University of China Beijing 100081 China; ^2^ The Brain Science Center Beijing Institute of Basic Medical Sciences Beijing 100850 China; ^3^ School of Basic Medical Sciences Anhui Medical University Hefei 230032 China; ^4^ Clinical Medicine Department of Medical College Yanbian University Yanji 133002 China; ^5^ School of Life Science & Research Center for Natural Peptide Drugs, Shaanxi Engineering & Technological Research Centre for Conservation & Utilization of Regional Biological Resources Yanan University Yanan 716000 China; ^6^ Key Laboratory of Neurology (Hebei Medical University) Ministry of Education Shijiazhuang 050000 China; ^7^ Department of Neuroimmunology and Antibody Engineering Beijing Institute of Basic Medical Sciences Beijing 100850 China; ^8^ Beijing Engineering Research Center of Food Environment and Public Health Minzu University of China Beijing 100081 China

**Keywords:** alzheimer's disease, IGF1, microglia, microglia‐astrocyte crosstalk, *Rack1*

## Abstract

Alzheimer's disease (AD) is the most common neurodegenerative disorder. Microglia make significant contributions to neuroinflammation and the progression of AD. However, the regulatory role of microglial activation and the communication between microglia and astrocytes in AD are largely unknown. Here, it is found that *Rack1* levels are elevated in microglia of patients with AD and AD model mice. The conditional knockout of *Rack1* in microglia reduced Aβ aggregation, alleviated neuroinflammation, and rescued cognitive impairments in AD model mice. Mechanistically, the knockout of *Rack1* in microglia decreased the number of microglia while increasing both the numbers and phagocytic activities of astrocytes by upregulating the levels of IGF1. The inhibition of IGF1R blocked microglial *Rack1* deficiency‐induced astrocyte proliferation and astrocyte‐mediated phagocytosis both in vitro and in vivo. Collectively, the results demonstrated that microglial *Rack1* contributes to AD pathology, at least partially through influencing IGF1‐IGF1R signaling between microglia and astrocytes, thus providing a potential target for AD treatment.

## Graphical abstract

Alzheimer's disease (AD) is the most common neurodegenerative disorder. This study demonstrates that microglial *Rack1* deficiency decreased Aβ deposition and neuroinflammation and ameliorated cognitive impairments in AD model mice by enhancing IGF1‐mediated astrocytic phagocytosis. The results provide insights into microglia‐astrocyte crosstalk and identify a potential therapeutic target for AD.

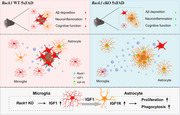

## 1 Introduction

Alzheimer's disease (AD) is characterized by extracellular amyloid beta (Aβ) deposition, intraneuronal tau hyperphosphorylation, and neuroinflammation, leading to synaptic loss, neuronal cell death, and, ultimately, cognitive impairment.^[^ ^1^, ^2^ ^]^ Despite AD being one of the most prevalent diseases among older people, the molecular mechanisms contributing to its pathophysiology are still not fully understood.

Microglia are the brain's resident immune cells, and play pivotal roles in the development of the central nervous system (CNS) and the maintenance of its homeostasis.^[^ ^3^, ^4^ ^]^ In AD pathology, when Aβ plaques are present in the brain, microglia proliferate, associate with the plaques, and engulf them, contributing to the slowing of disease progression.^[^ ^5^, ^6^, ^7^ ^]^ However, excessive microglial activation aggravates neuronal damage in advanced stages of amyloid pathology.^[^ ^8^, ^9^, ^10^ ^]^ The recent identification and subsequent validation of disease‐associated microglia (DAM),^[^ ^11^ ^]^ a previously unknown microglial type, suggests that microglia are more complex than previously thought.

We have previously shown that autophagy targets the NLRP3 inflammasome and mitochondrial antiviral signaling protein (MAVS), thus modulating microglia‐induced neuroinflammation and neurotoxicity.^[^ ^12^, ^13^ ^]^ Switching microglial metabolism from anaerobic glycolysis to mitochondrial oxidative phosphorylation was shown to ameliorate AD‐like pathology by enhancing microglial Aβ clearance.^[^ ^14^ ^]^ Moreover, calcium homeostasis modulator protein 2 (CALHM2) regulates calcium homeostasis and microglia‐mediated activation of inflammation, and, in an AD mouse model, the deletion of *Calhm2* in microglia leads to the attenuation of cognitive impairment.^[^ ^15^ ^]^ Although microglial activation and AD progression are highly correlated, the mechanisms underlying how microglia are activated remain largely unclear.

*Rack1*, belonging to the tryptophan‐aspartate (WD) repeat family of proteins, acts as a scaffold for different kinases and membrane receptors, thereby facilitating crosstalk between different signaling pathways. It plays a key role in a range of physiological processes, including development, aging, immune responses, cancer, and brain functions.^[^ ^16^, ^17^, ^18^, ^19^ ^]^ *Rack1* has been reported to act as a gatekeeper during p21‐driven neural stem cell (NSC) senescence, thus regulating the development of the cerebral cortex.^[^ ^20^ ^]^ Moreover, *Rack1* promotes the proliferation of THP‐1 cells by, at least in part, increasing GSK3β activity.^[^ ^21^ ^]^ *Rack1* also enhances self‐renewal and chemoresistance in cancer stem cells by interacting with and stabilizing Nanog.^[^ ^22^ ^]^ Notably, *Rack1* is highly expressed in neuronal cells of the CNS and contributes to the development of AD pathology through its contribution to Aβ production and Aβ‐induced toxicity.^[^ ^18^ ^]^ A recent study showed that *Rack1* is an essential component in the activation of the NLRP3 inflammasome in macrophages.^[^ ^23^ ^]^ Immune activation in response to inflammatory signals is a common phenomenon in AD, usually involving the assembly of the NLRP3 inflammasome and the activation of microglial cells. However, how *Rack1* contributes to microglial activation remains largely unknown.

In the present study, we aim to investigate the role of *Rack1* in microglial functions in AD pathology and sought to identify the underlying regulatory mechanism.

## 2 Results

### 2.1 *Rack1* Expression is Elevated in Microglia of AD Patients and AD Model Mice

**Figure 1:**
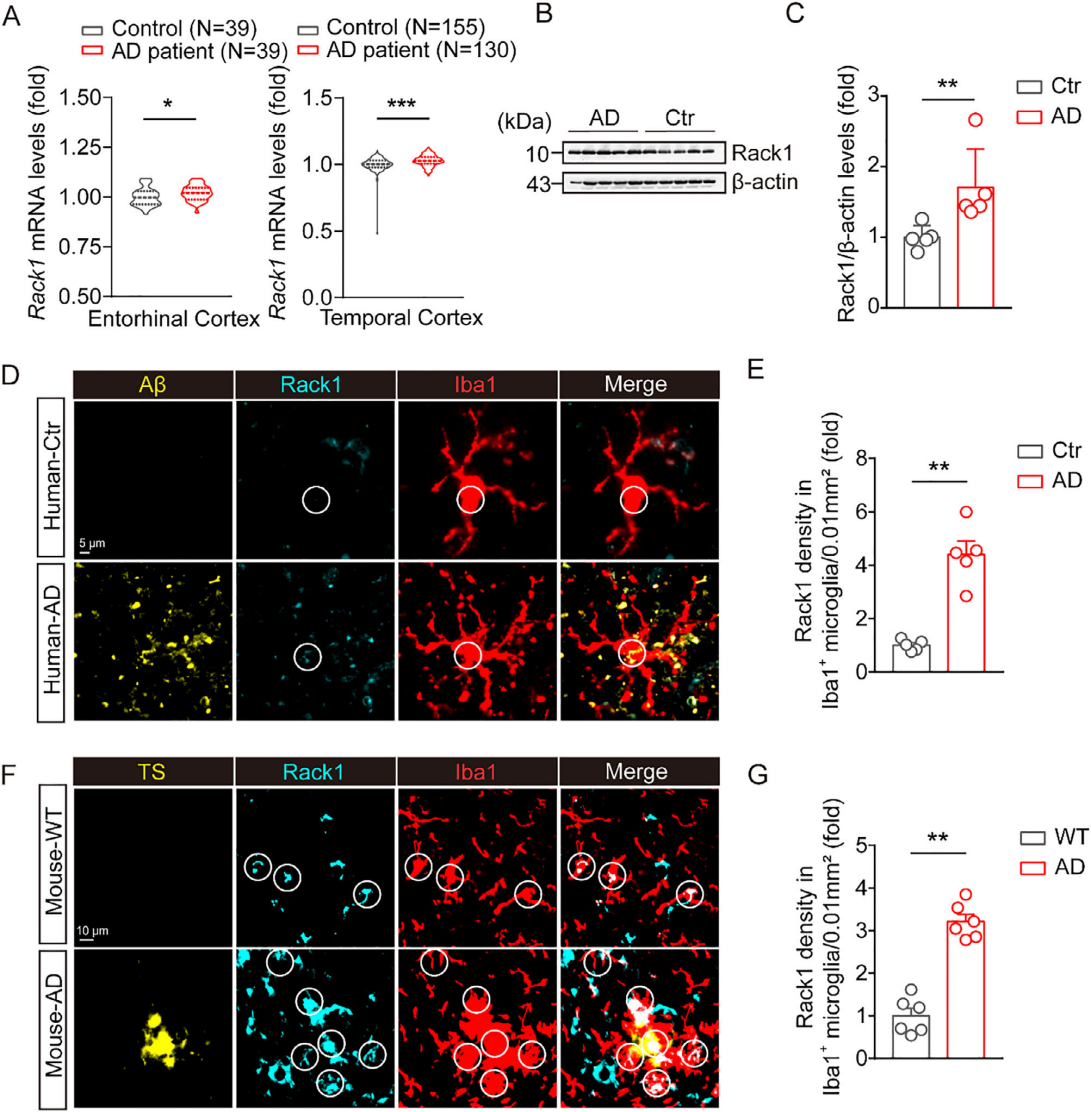
*Rack1* is increased in microglia of patients with AD and AD mice model. A) Increase of mRNA level of *Rack1* in entorhinal cortex (EC) tissue of AD patients (control, *N* = 39; AD patient, *N* = 39; GSE26927, GSE5281, and GSE48350), in temporal cortex (TC) tissue of patients with AD (control, *N* = 155; AD patient, *N* = 130, GSE5281, GSE15222, and GSE37263). The *Rack1* levels in normal control (NC) groups were normalized to 1, respectively, and then integrated into one group. B,C) Protein levels of *Rack1* in the hippocampal tissue samples from control and patients with AD (Ctr, *N* = 5; AD, *N* = 5). D,E) The quantification of levels of *Rack1* in microglia from control and patients with AD (Ctr, *N* = 5; AD, *N* = 5). F,G) The quantification of levels of *Rack1* in microglia from 6‐month‐old WT and *5×FAD* mice (WT, *n* = 6 mice; AD, *n* = 6 mice). All values are presented as mean ± SEM. ^*^ *p* < 0.05, ^**^ *p* < 0.01, and ^***^ *p* < 0.001. Mann‐Whitney U test (A, C, E, and G).

Analysis of our previously generated microglial RNA sequencing (RNA‐seq) data from 5×FAD model mice indicated that *Rack1* levels are upregulated in microglia of AD mice, showing an approximate 1.75 ± 0.06‐fold increase compared with their wild‐type (WT) counterparts (Figures S1A and S1B, Supporting Information). Similarly, *Rack1* expression was also found to be elevated in DAM clusters in a previously published database (GEO: GSE98969)^[^ ^11^ ^]^ (Figure S1C–F, Supporting Information). To further evaluate the potential contribution of *Rack1* to AD, we investigated how its levels change in AD patients using the AlzData web server (www.alzdata.org; GSE26927, GSE5281, and GSE48350 datasets). Our results revealed that, compared to healthy controls, *Rack1* levels were significantly elevated in entorhinal cortex samples of AD patients. Moreover, analysis of the GSE5281, GSE15222, and GSE37263 datasets indicated that *Rack1* levels were significantly upregulated in temporal cortex tissue (**Figure** **1**A). In line with this, we found that *Rack1* protein levels were significantly upregulated in hippocampal tissue of AD patients, as determined by western blotting (Figure 1B,C). Meanwhile, immunostaining for *Rack1* indicated that its expression levels were significantly higher in microglia of AD patients than in those of healthy controls (Figure 1D,E), with similar results obtained in 6‐month‐old 5×FAD mice (Figure 1F,G). Collectively, our results demonstrated that *Rack1* expression is elevated in patients with AD and AD model mice, suggesting that *Rack1* might play an important role in the development of this disorder.

### 2.2 The Conditional Knockout of *Rack1* in Microglia Ameliorates Cognitive Impairment in 5×FAD Mice

**Figure 2:**
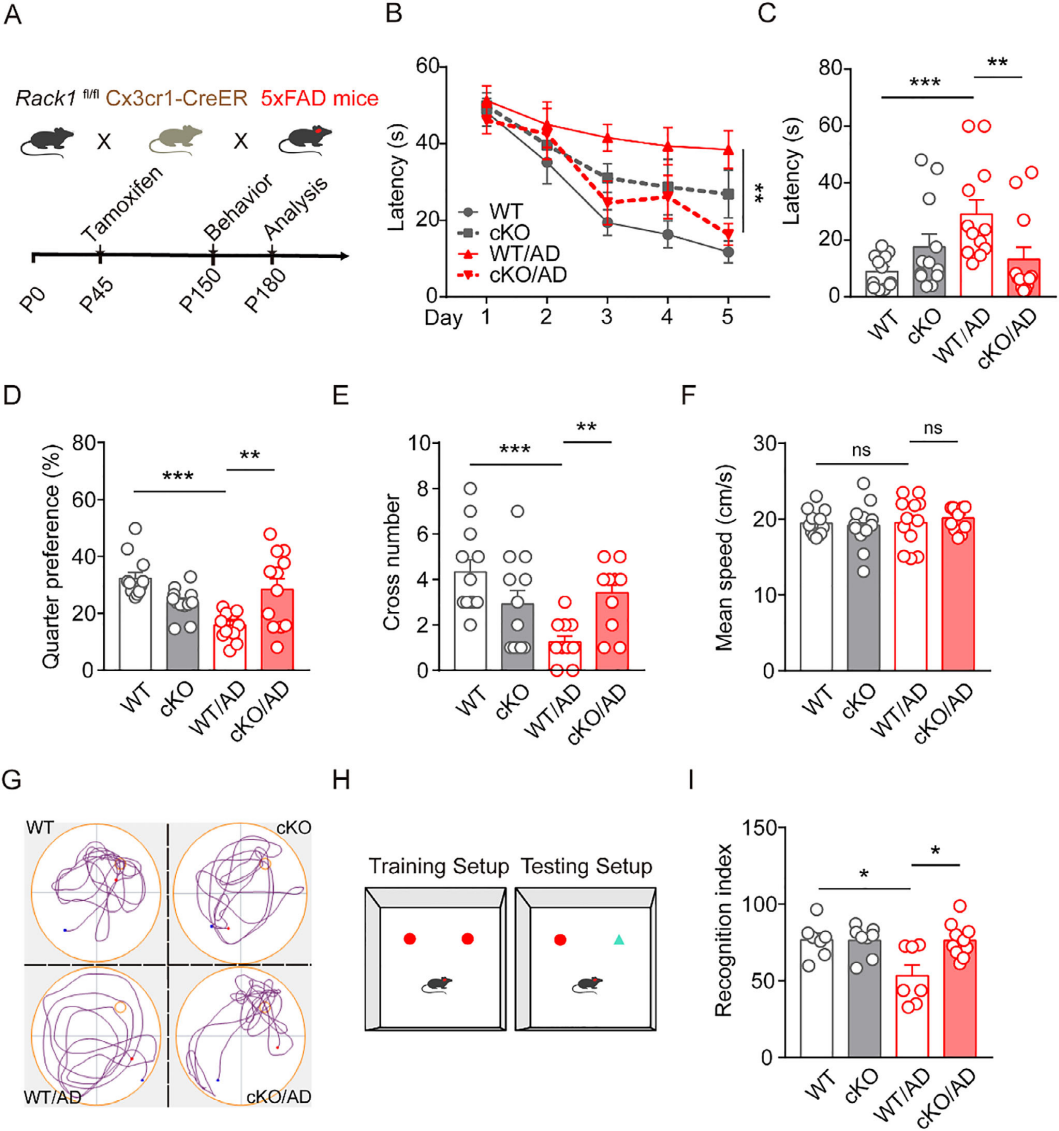
Conditional knockout of *Rack1* in microglia ameliorates cognitive impairment in 5×FAD mice. A) Schematic for the generation of mice, behavioral tests, and pathological analysis. B) MWM analysis of latency (s) to target in the platform training. C–F) MWM analysis of the latency (s), target quarter preference (%), target cross number, and mean speed (cm/s) in the platform tests in 5‐month‐old *Rack1* WT mice (n = 12), *Rack1* cKO mice (n = 12), *Rack1* WT/AD mice (n = 12), and *Rack1* cKO/AD mice (n = 12). G) Representative images of the track plots in the MWM tests. H) Schematic for the NOR test. I) Recognition index of mice in the NOR test in 5‐month‐old *Rack1* WT mice (n = 7), *Rack1* cKO mice (n = 8), *Rack1* WT/AD mice (n = 7), and *Rack1* cKO/AD mice (n = 10). All values are presented as mean ± SEM. ^*^ *p* < 0.05, ^**^ *p* < 0.01, and ^***^ *p* < 0.001. Two‐way ANOVA with Tukey's multiple comparisons test (B), and Kruskal–Wallis test (C–F,I).

Given that we found that *Rack1* levels were upregulated in microglia of AD patients and AD mice, we next constructed a *Rack1* conditional knockout AD mouse line by crossing *Rack1* ^flox/flox^, *Cx3cr1‐*CreER with 5×FAD transgenic mice (**Figure** **2**A). *Rack1* expression levels were assessed in all four groups of mice—*Rack1* WT (*Rack1* ^flox/flox^), *Rack1* cKO (*Rack1* ^flox/flox^ *^:Cx3cr1^* ^−CreER^), *Rack1* WT/AD (*Rack1* ^flox/flox^:5×FAD), and *Rack1* cKO/AD (*Rack1* ^flox/flox:^ *^Cx3cr1^* ^−CreER^:5×FAD)—at 6 months of age. The levels of *Rack1* were significantly reduced in microglia from *Rack1* cKO mice, but showed no significant change in astrocytes. The mRNA levels of *Rack1* were also significantly decreased in microglia isolated from *Rack1* cKO and *Rack1* cKO/AD mice, suggesting that knockout efficiency was high (Figure S2A–D, Supporting Information). Next, we assessed whether the conditional knockout of *Rack1* in microglia affected cognitive functions in mice using the Morris water maze (MWM) test. In the training period, *Rack1* WT/AD mice spent more time locating the hidden platform, indicative of impaired cognition. However, these cognitive deficits were significantly reversed in mice with conditional knockout of *Rack1* in microglia (*Rack1* cKO/AD mice) (Figure 2B). In the probe trial, compared with mice in the *Rack1* WT/AD group, *Rack1* cKO/AD mice demonstrated reduced latency to find the platform (Figure 2C), along with increases in the time spent in the target quarter (Figure 2D) and number of times crossing the platform location (Figure 2E); however, swimming speed was unchanged (Figure 2F,G). These findings suggested that the loss of *Rack1* in microglia alleviates cognitive impairment in AD mice. In line with the MWM results, in the novel object recognition (NOR) test, *Rack1* cKO/AD mice exhibited a greater preference for the novel object compared with *Rack1* WT/AD mice (Figure 2H,I), with no significant change in total interaction time (Figure S3A, Supporting Information). Together, these findings implied that the conditional knockout of *Rack1* in microglia ameliorates cognitive impairment in AD model mice.

### 2.3 The Conditional Knockout of *Rack1* in Microglia Decreases Aβ Deposition, Dendritic Spine Loss, and Microglial‐Mediated Phagocytic Activity

**Figure 3:**
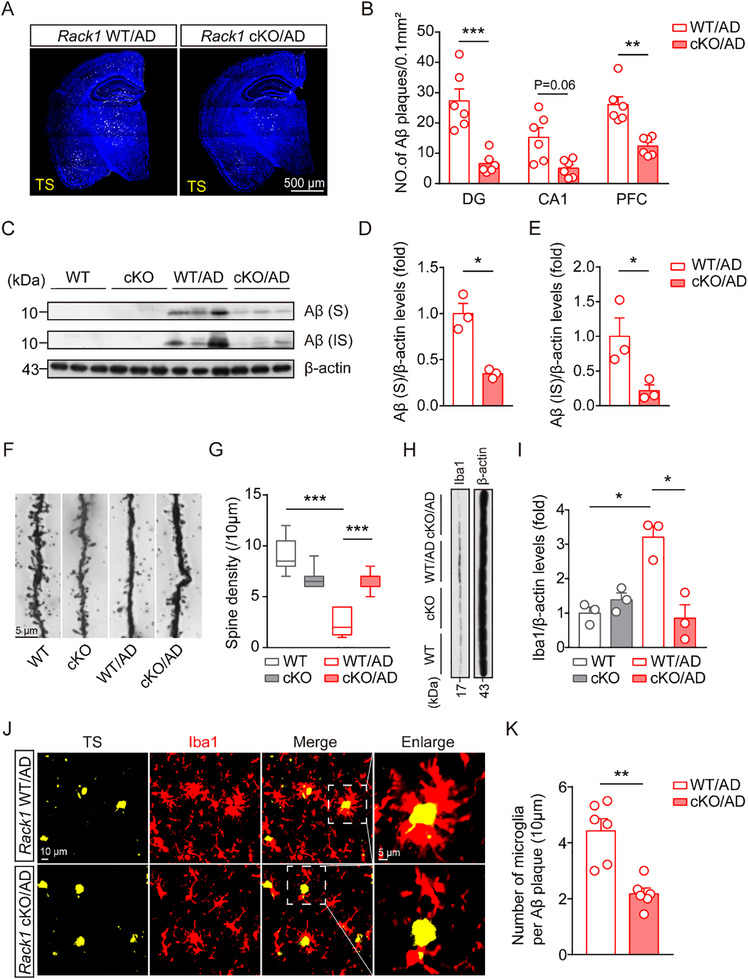
Conditional knockout of *Rack1* in microglia decreases Aβ deposition, spine loss, and microglial phagocytic activity. A,B) TS staining and statistical analysis of Aβ plaque numbers in DG, CA1, and PFC of 6‐month‐old *Rack1* WT/AD mice, and *Rack1* cKO/AD mice (n = 6 mice per group). C–E) Immunoblotting and statistical analysis of soluble (S) and insoluble (IS) Aβ levels of 6‐month‐old *Rack1* WT, *Rack1* cKO, *Rack1* WT/AD, and *Rack1* cKO/AD hippocampal tissue. F,G) Representative images and statistical analysis of dendritic spines using Golgi staining (n = 24 from 3 mice per group). H,I) Immunoblotting and statistical analysis of levels of Iba1 in 6‐month‐old *Rack1* WT mice, *Rack1* cKO mice, *Rack1* WT/AD mice, and *Rack1* cKO/AD mice (n = 3 mice per group). The protein levels were quantified by software Image J. Each point in the figure represents the relative protein level from one brain. J,K) TS staining, immunofluorescent staining of Iba1, and statistical analysis of microglia number per Aβ plaque in PFC of 6‐month‐old *Rack1* WT/AD mice and *Rack1* cKO/AD mice (n = 6 mice per group). All values are presented as mean ± SEM. ^*^ *p* < 0.05, ^**^ *p* < 0.01, and ^***^ *p* < 0.001. Two‐way ANOVA with Tukey's multiple comparisons test (B), Mann‐Whitney U test (D,E,K), and Kruskal–Wallis test (G,I).

Next, we evaluated whether the conditional knockout of *Rack1* in microglia altered Aβ levels in the AD mouse brain. As shown in **Figure** **3**A,B, the conditional loss of *Rack1* in microglia significantly reduced Aβ plaque accumulation in the hippocampal dentate gyrus (DG) and prefrontal cortex (PFC) regions, with a decreasing trend detected in the hippocampal CA1 region. Additionally, significant reductions in the contents of both soluble and insoluble Aβ were observed in *Rack1* cKO/AD mice relative to those in *Rack1* WT/AD mice (Figure 3C–E), suggesting that the conditional knockout of *Rack1* in microglia inhibited Aβ accumulation in the brain. Meanwhile, conditional *Rack1* deficiency in microglia did not alter the levels of APP, Nicastrin, or PSEN2, three key regulators in the Aβ production (Figure S4A–D, Supporting Information), suggesting that APP metabolism was unaffected.

To investigate the effects of the conditional knockout of *Rack1* in microglia on synapse plasticity, Golgi staining was employed to analyze the dendritic spine density in neurons of the hippocampal CA1 region. We found that the total dendritic spine density was significantly lower in *Rack1* WT/AD mice than in both *Rack1* WT and *Rack1* cKO/AD mice (Figure 3F,G), suggesting that the conditional knockout of *Rack1* in microglia may alleviate AD‐related synaptic damage in mice.

Given that we found that microglial *Rack1* knockout significantly reduced Aβ plaque accumulation in AD mice, we next investigated whether this phenomenon was due to changes in microglia. We observed that the knockout of *Rack1* significantly decreased the numbers of microglia in the DG, CA1 region, and PFC region of mice (Figure S5A,B, Supporting Information). Moreover, we found that Iba1 protein levels were also decreased in brain tissue of mice in the *Rack1* cKO/AD group (Figure 3H,I). We further evaluated the phagocytic activity of microglia using thioflavin‐S (TS) and Iba1 staining. The results showed that the knockout of *Rack1* in microglia significantly reduced the number of microglia surrounding Aβ plaques (Figure 3J,K), and these microglia exhibited reduced levels of triggering receptor expressed on myeloid cells 2 (TREM2, an important microglial phagocytic receptor^[^ ^24^ ^]^) and cluster of differentiation 68 (CD68) (Figure S5C–F, Supporting Information). This suggested that the decline in the Aβ plaque count may not have been directly due to microglia‐mediated phagocytosis.

To further confirm the effect of *Rack1* on microglia, we achieved stable knockdown of *Rack1* in BV2 cells, a mouse microglial cell line (Figure S6A, Supporting Information). The knockdown of *Rack1* in these cells significantly inhibited their proliferative and migratory activities (Figure S6B–D, Supporting Information). Moreover, *Rack1* knockdown significantly decreased the LPS‐induced production of proinflammatory cytokines, including IL‐1β, iNOS, and TNF‐α (Figure S6E–H, Supporting Information).

### 2.4 The Conditional Knockout of Microglial *Rack1* Increases Astrocyte Numbers and Astrocyte‐Mediated Aβ Engulfment

**Figure 4:**
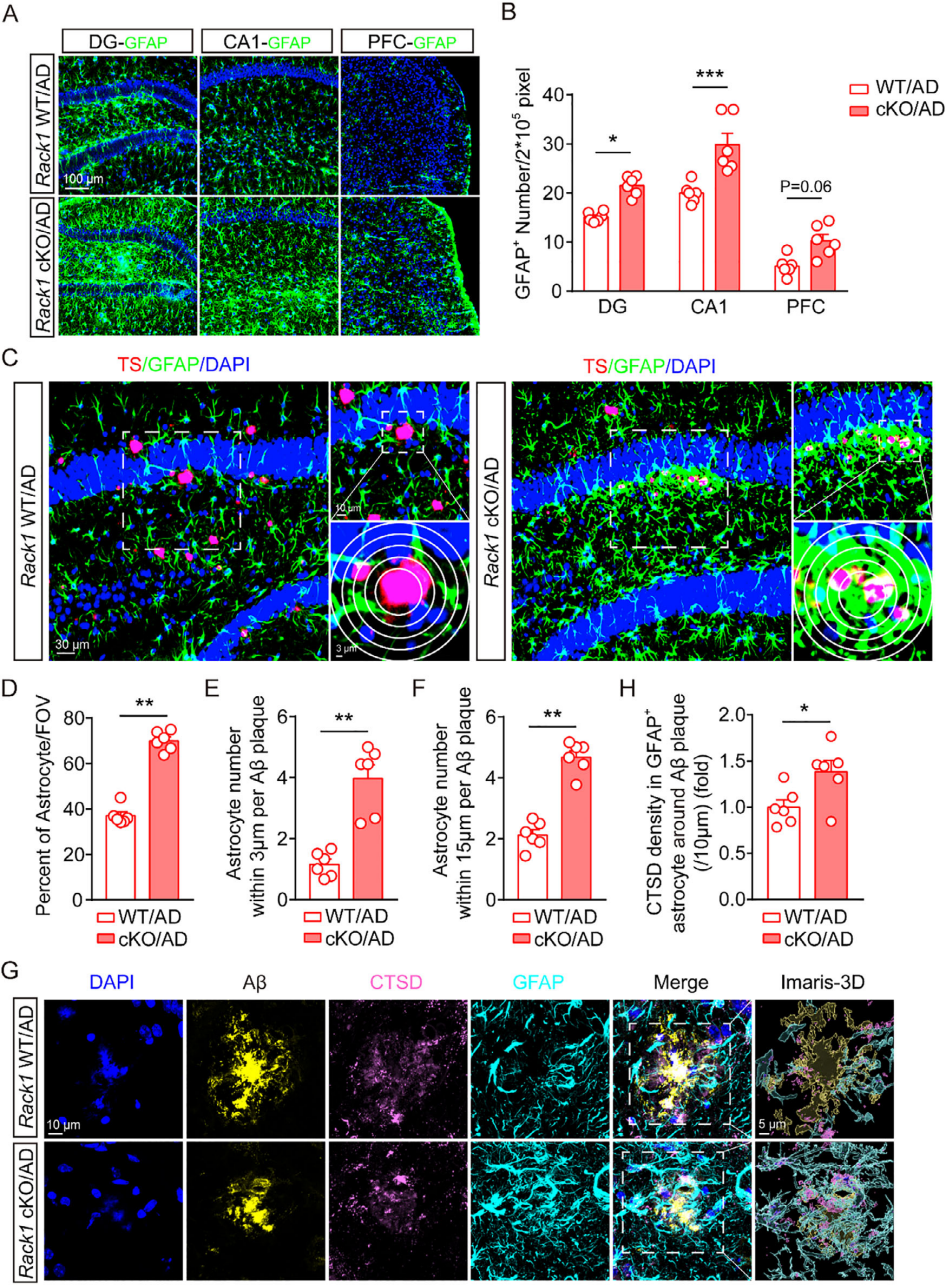
Conditional knockout of microglial *Rack1* increases astrocytic number and the engulfment of Aβ. A,B) Immunofluorescent staining and statistical analysis of GFAP‐positive cells in DG, CA1, and PFC of these four 6‐month‐old groups mice as indicated (n = 6 mice per group). C–F) TS staining, immunofluorescent staining of GFAP, and statistical analysis of percent of astrocyte in field of view, astrocyte number within 3 and 15 µm per Aβ plaque in hippocampus of 6‐month‐old *Rack1* WT/AD mice and *Rack1* cKO/AD mice (n = 6 mice per group). G,H) Immunofluorescent staining of Aβ, CTSD and GFAP, and statistical analysis of CTSD density in astrocyte around Aβ plaque in 6‐month‐old *Rack1* WT/AD mice and *Rack1* cKO/AD mice (n = 6 mice per group). All values are presented as mean ± SEM. ^*^ *p* < 0.05, ^**^ *p* < 0.01, and ^***^ *p* < 0.001. Two‐way ANOVA with Tukey's multiple comparisons test (B), and Mann–Whitney U test (D–F,H).

Astrocyte activation is also involved in Aβ plaque clearance, and communication between microglia and astrocytes plays an important role in the pathology of AD.^[^ ^25^, ^26^, ^27^, ^28^, ^29^ ^]^ Accordingly, we subsequently examined whether astrocyte changes contribute to this process. Notably, we found that the number of astrocytes in the hippocampal DG and CA1 regions was significantly increased in *Rack1* cKO/AD mice when compared with *Rack1* WT/AD mice, with an increasing trend also detected in the PFC region (**Figure**  **4**A,B). Next, we performed Ki‐67 staining, one protein that widely used as a proliferation marker. We found that the number of Ki67‐positive astrocytes was also increased in *Rack1* cKO/AD mice brains (Figure S7A,B, Supporting Information), suggesting that microglial *Rack1* knockout leads to an increase in astrocyte numbers in the brains of AD mice.

To further investigate whether the increase in the number of astrocytes contributed to the observed reduction in Aβ deposition in *Rack1* cKO/AD mice, we employed TS and GFAP staining. We found that microglial *Rack1* knockout increased not only the total number of astrocytes, but also the number of astrocytes surrounding Aβ plaques (Figure 4C–F). We also found that the levels of cathepsin D (CTSD, a lysosomal protease whose contents reflect degradative activity) were significantly increased in astrocytes of *Rack1* cKO/AD mice (Figure 4G,H). Collectively, these findings demonstrate that the knockout of *Rack1* in microglia increases Aβ phagocytosis in astrocytes.

### 2.5 Transcriptome Analysis Revealed that the Knockout of *Rack1* in Microglia Increases Astrocyte Proliferation and Phagocytic Activity

**Figure 5:**
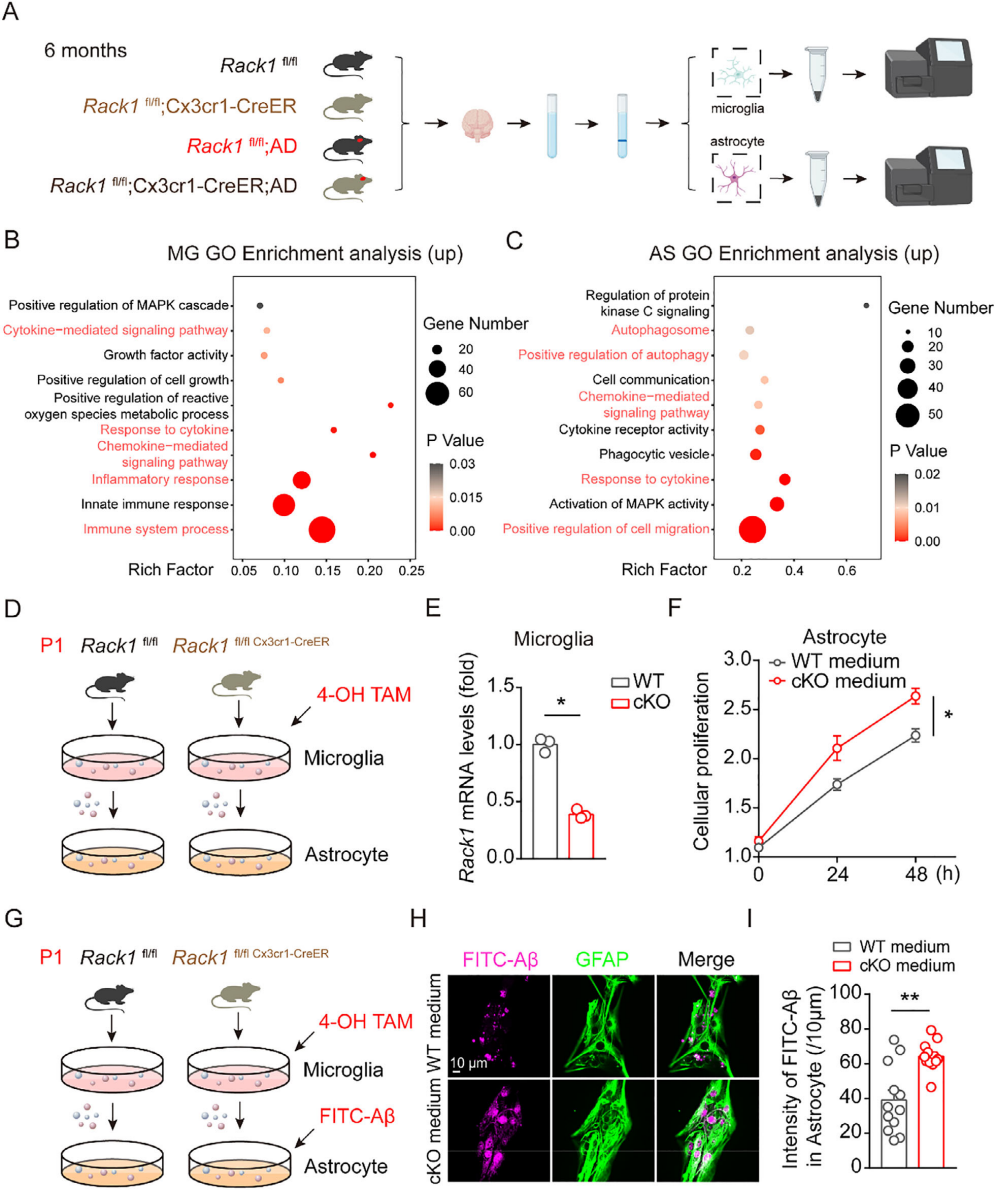
Transcriptome changes reveal knockout of *Rack1* in microglia increases astrocytic proliferation and phagocytosis. A) Schematic for microglia and astrocyte isolation, RNA extraction, RNA‐seq, and bioinformatic analysis. B,C) GO enrichment analysis of microglia and astrocyte shows two groups of upregulated expressed genes between *Rack1* WT/AD mice and *Rack1* cKO/AD mice (log_2_ fold change ≥ 0.5, adjusted false discovery rate < 0.05). D) Isolating primary microglia and astrocyte from P0‐P3 of *Rack1* WT mice, *Rack1* cKO mice, and treat 4‐OH for 4 days. E,F) Transcriptional levels of *Rack1* in isolated primary microglia, and the effect of microglial conditional medium in astrocyte using CCK8 assay. G) Schematic for phagocytosis of FITC‐Aβ assay using microglial conditional medium. H,I) Immunofluorescent staining of GFAP, and statistical analysis of FITC‐Aβ intensity in astrocyte. All values are presented as mean ± SEM. ^*^ *p* < 0.05, ^**^ *p* < 0.01, and ^***^ *p* < 0.001. Mann–Whitney U test (E,I), and Two‐way ANOVA with Tukey's multiple comparisons test (F).

To further reveal the underlying molecular mechanisms, primary microglia and primary astrocytes were isolated from the brains of mice and were subsequently subjected to RNA‐seq analysis (**Figure** **5**A). A total of 863 genes were found to be upregulated and 1147 downregulated in microglia of *Rack1* cKO/AD mice when compared with *Rack1* WT/AD mice. In astrocytes, meanwhile, 3020 genes were upregulated and 1804 downregulated in the *Rack1* cKO/AD group relative to animals in the *Rack1* WT/AD group (Figure S8A,B, Supporting Information). Gene Ontology (GO) analysis of the significantly upregulated genes in *Rack1* cKO/AD mice revealed that they were enriched in the cytokine‐mediated signaling pathway. Additionally, pathways related to immune system processes, inflammatory responses, chemokine‐mediated signaling, and responses to cytokines were significantly upregulated in microglia of *Rack1* cKO/AD mice (Figure 5B). Next, Kyoto Encyclopedia of Genes and Genomes (KEGG) pathway enrichment analysis revealed that the genes exhibiting upregulation in microglia of *Rack1* cKO/AD mice were significantly associated with pathways such as cytokine‐cytokine receptor interaction and endocrine resistance (Figure S8C, Supporting Information). Meanwhile, the upregulated genes in astrocytes of the same group of mice were significantly enriched in pathways such as response to cytokine and chemokine‐mediated signaling, positive regulation of autophagy and autophagosome, positive regulation of cell migration (Figure 5C), cytokine‐cytokine receptor interaction, endocrine resistance, phagosome, and endocytosis (Figure S8D, Supporting Information). These results collectively suggested that the protective effects of microglial *Rack1* deficiency in AD pathology might be mediated via cytokine‐cytokine receptor interactions between microglia and astrocytes.

To confirm this possibility, primary microglia were isolated from both *Rack1* WT and *Rack1* cKO mice, and then treated with 4‐hydroxytamoxifen (4‐OH‐TAM) to knockout *Rack1*. The microglial conditioned medium was then transferred to primary astrocytes, and changes in astrocyte proliferation were recorded (Figure 5D). First, we found that *Rack1* levels were significantly decreased in *Rack1* cKO primary microglia, thus confirming *Rack1* knockout (Figure 5E). We further observed that the conditioned medium from *Rack1* cKO microglia significantly increased primary astrocyte viability compared with that from *Rack1* WT microglia (Figure 5F). Moreover, conditioned medium from *Rack1* cKO microglial culture also significantly increased the ability of astrocytes to phagocytose FITC‐Aβ (Figure 5G–I). As “autophagosome” was one of the enriched GO terms for upregulated genes in *Rack1* cKO/AD mice, and autophagosome formation plays a critical role in Aβ degradation, we checked whether autophagosome formation was affected in the AD mouse brain. The co‐staining of GFAP and TS/Aβ with microtubule‐associated protein 1A/1B‐light chain 3 (LC3) or phosphorylated autophagy‐related Gene 16‐Like 1 (p‐Atg16L1), two key autophagic markers, showed that microglial *Rack1* knockout upregulated the staining intensity of LC3 and p‐Atg16L1 in astrocytes surrounding Aβ plaques in the AD mouse (Figure S8E–H, Supporting Information). Collectively, these findings demonstrated that *Rack1* deficiency in microglial cells enhances the proliferative, phagocytic, and autophagic capacity of astrocytes through secreted factors.

### 2.6 The Effect of Microglial *Rack1* Deficiency on Astrocyte Proliferation and Phagocytic Activity is Mediated by IGF1

**Figure 6:**
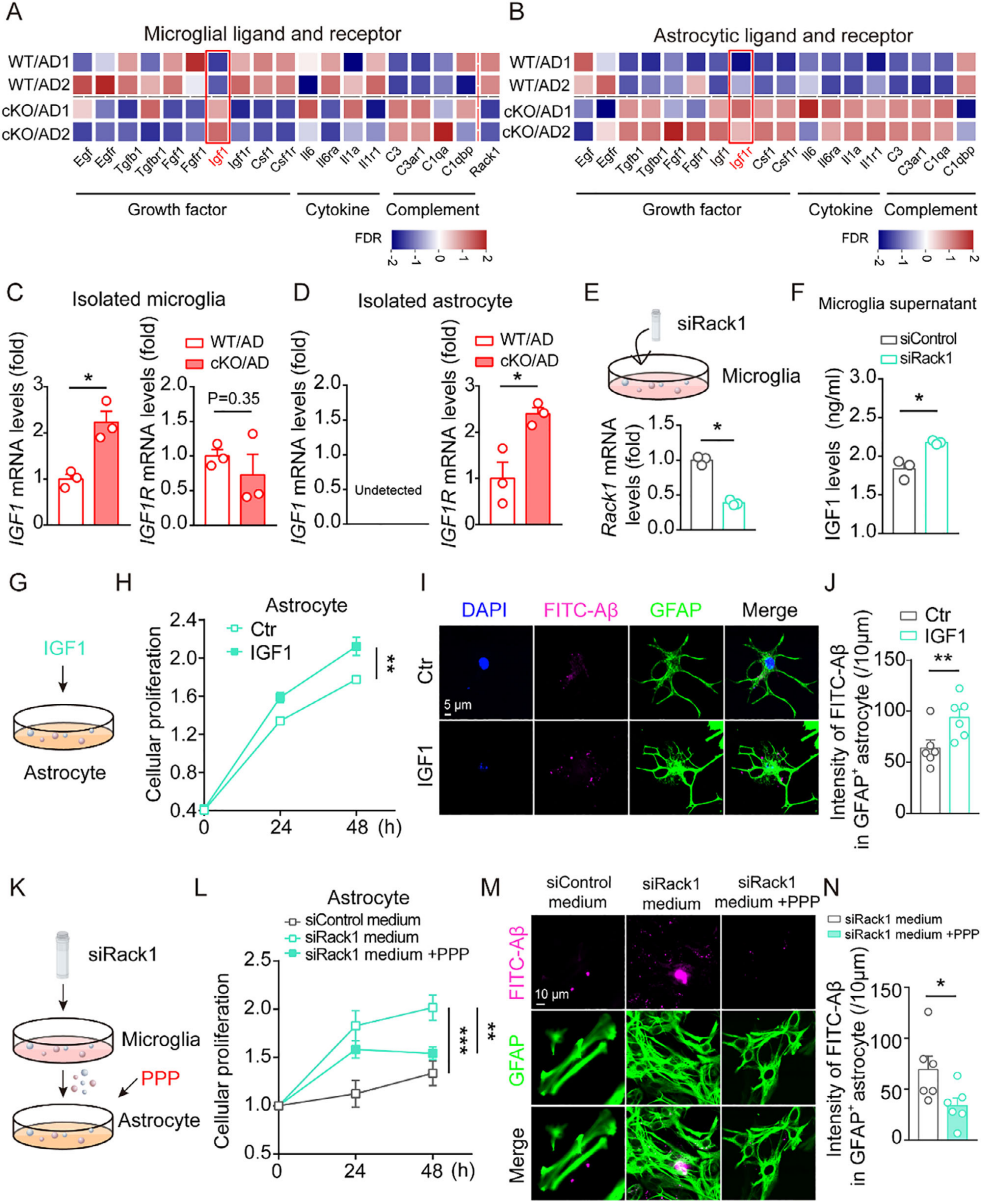
The role of *Rack1* deficiency in astrocytic proliferation and phagocytosis is mediated by IGF1. A) Heatmap of growth factors, cytokines, complement, and receptors in microglia from two groups of mice as indicated. B) Heatmap of growth factors, cytokines, complement, and receptors in astrocyte. C,D) qPCR assay to test the expression levels of IGF1 and IGF1R in isolated microglia and isolated astrocyte from 6‐month‐old *Rack1* WT/AD mice and *Rack1* cKO/AD mice (n = 3 per group). E) Schematic for siRack1 in primary microglia, and analysis of the knockdown efficiency. F) IGF1 Elisa kit to test IGF1 levels in microglia supernatant. G,H) Schematic for IGF1 to treat astrocyte and the effect of IGF1 in astrocyte using CCK8 assay (n = 3). I,J) Immunofluorescent staining of GFAP, and statistical analysis of FITC‐Aβ intensity in astrocyte with or without IGF1 treatment. K–N) Schematic for PPP treatment in astrocyte and test the effect of knockdown of *Rack1* in microglia using CCK8 assay (n = 4), phagocytosis of FITC‐Aβ (n = 6) in astrocyte with or without PPP treatment. All values are presented as mean ± SEM. ^*^ *p* < 0.05, ^**^ *p* < 0.01, and ^***^ *p* < 0.001. Mann–Whitney U test (C–F,J,N), and Two‐way ANOVA with Tukey's multiple comparisons test (H,L).

To identify the potential factors involved in these effects of *Rack1* deficiency, we analyzed the levels of growth factors, cytokines, and their receptors in both microglia and astrocytes. The expression levels of most of the evaluated growth factors (*Egf*, *Tgfb1*, *Fgf1*, and *Csf1*) and cytokines (*Il6*, *Il1a*, *Ccl3*, *Ccl4*, *Il1b*, and *Nos2*) were downregulated in microglia of the *Rack1* cKO/AD group, whereas that of the growth factor IGF1 was significantly upregulated (**Figures** **6**A; S9A, Supporting Information). Interestingly, the growth factor receptor IGF1R was also significantly upregulated in astrocytes of the *Rack1* cKO/AD group (Figure 6B). IGF1 is a hormone that mediates growth hormone (GH)‐stimulated physical growth. It has been reported that IGF1 modulates the activity of the mTOR pathway through its interaction with IGF1R.^[^ ^30^ ^]^ We further isolated microglia and astrocytes from both *Rack1* WT/AD and *Rack1* cKO/AD mice, and assessed the changes in IGF1 and IGF1R contents. We observed that the levels of IGF1 were significantly increased in microglia of *Rack1* cKO/AD mice, whereas those of IGF1R were unchanged. Interestingly, IGF1R levels were significantly elevated in astrocytes of the *Rack1* cKO/AD group of mice, whereas those of IGF1 were undetectable (Figure 6C,D). Additionally, the expression of *Ccl3*, *Ccl4*, *Il1β*, and *Nos2* was found to be decreased in *Rack1* cKO/AD mice (Figure S9B, Supporting Information). These results suggested that IGF1 might be involved in this process.

To confirm this hypothesis, we measured IGF1 levels in the microglial supernatant by enzyme‐linked immunosorbent assay (ELISA) and found that they were indeed increased in *Rack1*‐deficient primary microglia (Figure 6E,F). To test whether this effect of IGF1 was direct, we added IGF1 to the medium of cultured primary astrocytes, and found that IGF1 treatment enhanced both the viability (Figure 6G,H) and the FITC‐Aβ‐engulfing ability of astrocytes (Figure 6I,J). To test whether this effect was mediated by IGF1R in astrocytes, we applied an IGF1R inhibitor (picropodophyllin, PPP) and found that the enhancement of the proliferative and phagocytic abilities of astrocytes induced by microglial *Rack1* deficiency was weakened with PPP pretreatment (Figure 6K–N). Collectively, these results demonstrated that IGF1‐IGF1R signaling is involved in astrocyte proliferation and astrocyte‐mediated phagocytosis in mice with microglial *Rack1* deficiency.

### 2.7 The Inhibition of IGF1R Abolishes the Protective Role of Microglial *Rack1* Deficiency in 5×FAD Mice

**Figure 7:**
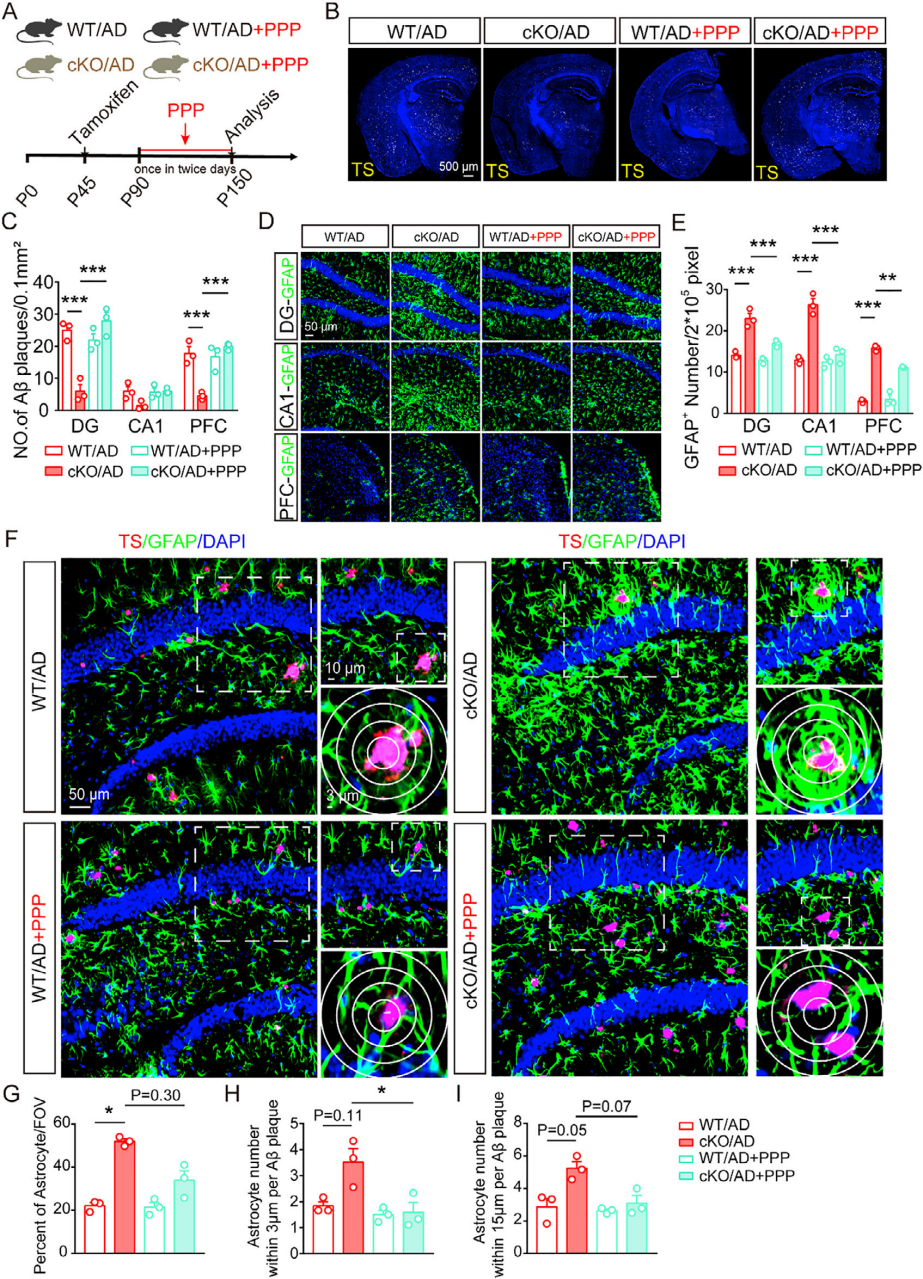
Inhibition of IGF1R abolishes the protective role of microglial *Rack1* deficiency in *5×FAD* mice. A) Schematic for intraperitoneal PPP injections in 5‐month‐old W *Rack1* WT/AD mice and *Rack1* cKO/AD mice. B,C) TS staining and statistical analysis of Aβ plaque numbers in DG, CA1, and PFC of 5‐month‐old of these four group mice as indicated (n = 3 mice per group). D,E) Immunofluorescent staining and statistical analysis of GFAP‐positive cells in DG, CA1, and PFC of 5‐month‐old these four group mice as indicated (n = 3 mice per group). F–I) TS staining, immunofluorescent staining of GFAP, and statistical analysis of the percent of astrocyte in the field of view, astrocyte number within 3 and 15 µm per Aβ plaque in hippocampal of 5‐month‐old *Rack1* WT/AD mice, *Rack1* cKO/AD mice, *Rack1* WT/AD+PPP mice, and *Rack1* cKO/AD+PPP mice (n = 3 mice per group). All values are presented as mean ± SEM. ^*^ *p* < 0.05, ^**^ *p* < 0.01, and ^***^ *p* < 0.001. Two‐way ANOVA with Tukey's multiple comparisons test (C,E), and Kruskal–Wallis test (G–I).

To determine whether IGF1‐IGF1R signaling is involved in AD pathology in vivo, we administered the IGF1R inhibitor PPP to both *Rack1* WT/AD and *Rack1* cKO/AD mice via intraperitoneal injection once every 2 days for 2 months (**Figure** **7**A). We found that while the conditional knockout of *Rack1* in microglia greatly reduced the number of Aβ plaques in the hippocampus and cortex, this effect was markedly blocked following PPP administration (Figure 7B,C). This suggested that IGF1‐IGF1R signaling contributes to the decrease in Aβ plaque numbers. Next, we evaluated whether astrocytes are involved in this process. We observed that the number of astrocytes in the DG, CA1, and PFC brain areas was noticeably higher in *Rack1* cKO/AD mice than in their *Rack1* WT/AD counterparts. However, PPP treatment significantly reversed these effects (Figure 7D,E). Moreover, PPP treatment also largely decreased the number of astrocytes surrounding Aβ plaques in *Rack1* cKO/AD mice (Figure 7F–I). These observations suggested that suppressing IGF1R resulted in a decline in both total astrocyte numbers and the number of astrocytes surrounding Aβ plaques. Next, we investigated whether the administration of PPP also affected Aβ degradation in astrocytes, and found that treatment with PPP also decreased LC3 immunostaining intensity in astrocytes around Aβ plaques in AD mice (Figure S10A,B, Supporting Information). This suggested that blocking IGF1R inhibited microglial *Rack1* deficiency‐induced astrocyte proliferation, along with astrocyte‐mediated phagocytosis and autophagy. Moreover, we examined whether PPP treatment affected microglia in this process. We found that PPP treatment did not significantly affect total microglial numbers in the DG, CA1, and PFC (Figure S11A,B, Supporting Information). Interestingly, there were fewer TREM2 contents in microglia surrounding Aβ plaques in the brains of *Rack1* WT/AD+PPP mice than in those of *Rack1* WT/AD animals, accompanied by a decreased trend of microglia number and CD68 contents (Figure S11C–H, Supporting Information). This implied that inhibiting IGF1R slightly decreases the microglia‐mediated phagocytosis of Aβ plaques.

Finally, we investigated whether IGF1R inhibition would abolish the cognitive benefits associated with *Rack1* deficiency in microglia. Treatment with the IGF1R inhibitor PPP reversed the cognitive improvement observed in AD mice with microglial *Rack1* knockout in the MWM and NOR tests (Figure S12A–E, Supporting Information). Together with the effects of IGF1R inhibition on AD pathology, these results implied that *Rack1* deficiency exerts its neuroprotective effects via the IGF1R pathway.

## 3 Discussion

Microglia are the predominant immune cells in the CNS, and their functions are critical in the pathophysiology of AD.^[^ ^31^, ^32^ ^]^ However, the regulatory mechanism underlying microglial activation and Aβ phagocytosis is still unclear. In this study, we found that *Rack1* levels were elevated in microglia of both patients with AD and AD model mice. The conditional deletion of microglial *Rack1* in the 5×FAD mouse model significantly reduced Aβ accumulation and alleviated cognitive impairment. Mechanistically, we found that microglial *Rack1* deletion decreased microglial numbers and inhibited microglia‐mediated Aβ phagocytosis, while simultaneously enhancing the proliferative and phagocytotic capacity of astrocytes. Further studies showed that the protective role of microglial *Rack1* deficiency in AD was mediated by IGF1‐IGF1R signaling between microglia and astrocytes, highlighting an important regulatory pathway in glial activation within AD pathology.

*Rack1* is a multifaceted scaffolding protein highly expressed in neuronal cells, and plays important roles under both physiological and pathological conditions.^[^ ^16^, ^17^, ^18^, ^19^ ^]^ Most studies of *Rack1* in AD have focused on neurons, where it has been reported to regulate APP processing and modulate Aβ‐induced toxicity.^[^ ^18^ ^]^ However, the role of *Rack1* in microglia in the CNS remains largely unknown. It has been shown that the knockdown of *Rack1* in microglia in vitro decreased the production of LPS‐induced pro‐inflammatory cytokines.^[^ ^33^ ^]^ Moreover, *Rack1* was identified as a component of NLRP3 complexes in macrophages, playing an essential role in NLRP3 inflammasome activation.^[^ ^23^ ^]^ Furthermore, NLRP3 deficiency in APP/PS1 mouse models was shown to reduce Aβ deposition, skew microglial cells toward an M2 phenotype, and alleviate cognitive impairment,^[^ ^34^ ^]^ suggesting that *Rack1* might also be involved in microglial activation and AD pathology. We found that the deletion of *Rack1* in microglia also reduced Aβ accumulation and attenuated cognitive impairments in AD model mice. However, microglial *Rack1* knockout decreased the total number of microglia as well as the number of microglia surrounding Aβ plaques in the AD mouse brain. This phenotype differs from that previously reported for *NLRP3* knockout, where *NLRP3* depletion increased the numbers of microglia surrounding Aβ plaques and enhanced microglia‐mediated phagocytosis. These observations suggest that the protective role of microglial *Rack1* inhibition in AD pathology might be independent of the NLRP3 inflammasome. Moreover, in this study, we found that knockdown of *Rack1* in BV2 cells significantly inhibited their proliferative and migratory activities and LPS‐induced production of proinflammatory cytokines, including IL‐1β, iNOS, and TNF‐α. Meanwhile, transcriptome analysis of *Rack1*‐deficient microglia in AD pathology showed that the production of cytokines, including Il6, Il1a, Ccl3, Ccl4, Il1β, and Nos2, were downregulated in the *Rack1* cKO/AD group. The reduction of these typical pro‐inflammatory cytokines suggests the downregulation of the pro‐inflammatory response in *Rack1* deficiency microglia. In contrast, we found that the up‐regulated cytokine‐mediated signaling pathways, immune system processes, inflammatory responses, and chemokine‐mediated signaling in microglia of *Rack1* cKO/AD mice. For example, genes involved in immune response pathways, including Ccl12, Ccl8, Arg1, Cxcl13, and Ccl22, were significantly upregulated, indicating the feedback regulatory mechanism might be involved. Moreover, we found that the growth factor Igf1 level was also significantly increased, and IGF1‐IGF1R signaling between microglia and astrocytes mediated the neuroprotection effect of *Rack1* deficiency in AD pathology. Together, these results suggest that *Rack1* deficiency inhibits the typical pro‐inflammatory response, but increases cytokine‐mediated signaling pathways.

Microglia and astrocytes are major glial cell types in the brain. The crosstalk between microglia and astrocytes is an important determinant of their functions, and plays a fundamental role in neuronal functions and multiple diseases.^[^ ^25^, ^26^, ^27^, ^28^, ^29^ ^]^ As the primary immune cells in the CNS, microglia typically react faster than astrocytes to brain damage stimuli, initiating astrocyte activation and controlling astrocyte functions.^[^ ^35^, ^36^, ^37^ ^]^ Concurrently, astrocytes serve as supportive glial cells in the brain and are involved in neuroinflammation and shaping microglial functions. The co‐occurrence of microgliosis and astrocytosis in AD pathology further suggests that microglia‐astrocyte interactions play a pivotal role in this process. For instance, CCL2‐CCR2 signaling between microglia and astrocytes is implicated in neuroinflammation, with its upregulation causing cognitive dysfunction.^[^ ^38^ ^]^ Moreover, astrocyte‐derived IL‐3 reprograms microglia to increase IL‐3Rα expression, boosting the capacity of microglia to clear Aβ and tau protein aggregates, thereby improving AD pathology.^[^ ^39^ ^]^ Meanwhile, complement‐dependent intercellular crosstalk has also been identified, where astrocytic NF‐κB signaling elicits the extracellular release of C3. This C3, in turn, interacts with neuronal and microglial C3aR, promoting a pathogenic cycle that alters neuroinflammation and AD pathology.^[^ ^36^ ^]^

In this study, we discovered that microglial *Rack1* deficiency increases IGF1 expression in microglia, which subsequently enhances astrocyte proliferation and phagocytosis. Importantly, blocking IGF1‐IGF1R signaling reversed the effects of *Rack1* deficiency on AD pathology in mice, thereby identifying a novel critical signaling pathway in microglia‐astrocyte crosstalk and suggesting a potential therapeutic target for AD treatment.

IGF1 is a neurotrophic hormone with critical roles in a wide range of biological activities, including CNS development, maturation, and disorders.^[^ ^40^, ^41^ ^]^ Clinical studies have shown that low IGF1 levels are closely related to a higher risk of incident AD, increased brain Aβ deposition, and cognitive decline.^[^ ^42^, ^43^, ^44^, ^45^ ^]^ In the brain, IGF1 plays a neuroprotective role by promoting neurogenesis, neuronal differentiation, and synapse formation.^[^ ^46^, ^47^ ^]^ It is reported that circulating IGF1 mediates exercise‐induced neurogenesis in the adult hippocampus.^[^ ^48^ ^]^ Furthermore, increased IGF1 levels also contribute to enhanced neurogenesis, improved memory accuracy, and delayed neuronal degeneration, thus ameliorating age‐related cognitive dysfunction.^[^ ^49^, ^50^ ^]^ Experiments in mouse models have also shown that IGF1 can improve cognitive function.^[^ ^51^, ^52^ ^]^ Specifically, IGF1 administration in 24‐month‐old male mice improved learning and memory and reduced depression and anxiety‐like behaviors.^[^ ^52^ ^]^ Here, we found that the microglial conditional knockout of *Rack1* increased IGF1 levels in microglia, which further enhanced the proliferation of astrocytes and their phagocytic activity. This ultimately led to a reduction in neuroinflammation, less Aβ plaque formation, and improved cognitive functions in AD model mice. Considering the close association between IGF1 levels and cognitive functions in clinical settings, our study reveals a new regulatory mechanism for IGF1 expression and function in AD pathology, thus providing a novel strategy for AD treatment.

However, some interesting questions remain: i) How does *Rack1* regulate IGF1 expression in microglia? Our data showed that the knockout of *Rack1* increased the transcript levels of IGF1 in primary microglia; however, the underlying mechanism remains unknown. It has been reported that *Rack1* directly interacts with IGF1R and regulates its kinase activity.^[^ ^53^ ^]^ This suggests that *Rack1* deficiency decreases IGF1R activity in microglia, thereby inducing IGF1 upregulation via an unknown feedback pathway, a possibility that warrants further investigation. ii) Are there other cell types involved in *RACK1*‐influenced IGF1‐IGF1R signaling in AD pathology? In our study, we found that the administration of an IGF1R inhibitor reversed the microglial *Rack1* deficiency‐induced protective effect of IGF1 in AD model mice, likely by inhibiting astrocyte proliferation and astrocyte‐mediated phagocytosis. However, we cannot exclude that IGF1R may be acting via neurons or other cell types.

In this study, we demonstrated that microglial *Rack1* deficiency decreased Aβ deposition and neuroinflammation and ameliorated cognitive impairments in AD model mice by enhancing IGF1‐mediated astrocytic phagocytosis. Our results provide insights into microglia‐astrocyte crosstalk and identify a potential therapeutic target for AD.

## 4 Experimental Section

### Human Samples

Human brain samples were obtained from the National Human Brain Bank for Development and Function, Chinese Academy of Medical Sciences and Peking Union Medical College, Beijing, China (approved by the Institutional Review Board of the Institute of Basic Medical Sciences, Chinese Academy of Medical Sciences; Approval Numbers: 009‐2014 and 2 022 125). The criteria for AD diagnosis were based on two previous studies,^[^ ^54^, ^55^ ^]^ and the operational protocol was as previously described.^[^ ^56^ ^]^ Detailed information is presented in Table S1 (Supporting Information).

### Mice

*Rack1* ^flox/flox^ mice were provided by Prof. Jiyan Zhang (Beijing Institute of Basic Medical Sciences, Beijing, China). The 5×FAD mice and *Cx3cr1*‐CreER mice used in this study were previously described.^[^ ^15^ ^]^ A total dose of 20 mg of TAM (Sigma–Aldrich, catalog no. T5648, in corn oil) was intragastrically administered to mice at day 45 for 5 consecutive days. Heterozygous *Cx3cr1*‐CreER mice were used in the study. Both male and female mice were chosen for the behavioral tests, and equal numbers of age‐ and gender‐matched mice from each group were chosen for the pathologic analysis. All animal experiments in this study were approved by the Institutional Animal Care and Use Committee of the Beijing Institute of Basic Medical Sciences (IACUC‐DWZX‐2019‐504).

### MWM Test

The MWM test was conducted as previously detailed.^[^ ^57^ ^]^ Briefly, mice were trained twice a day for 5 consecutive days, with the platform hidden and in the presence of spatial cues. On day 6, the target platform was removed, and the mice were introduced to the maze still in the presence of spatial cues. The original location of the platform was marked as the target. The latency to find the escape platform, the number of platform crossings, quarter preference, and average speed were recorded and analyzed.

### NOR Test

The NOR test was conducted as previously described.^[^ ^57^ ^]^ The experiment was carried out in a white rectangular box (50 × 50 × 20 cm) and was divided into three phases: acclimation, familiarization, and testing. On the first day, each mouse was placed in the empty box and allowed to freely explore for 5 min. On day 2, two identical objects were placed in the box, and the mice were left to freely explore the box for 5 min (first trial). After 1 h, the second trial was performed, with one of the original objects replaced with a novel object. Mice were again allowed to freely explore the box for 5 min. The time the mice spent exploring the original and novel objects was recorded. Mice that did not reach a minimum exploration time of 20 s were excluded from the analysis.^[^ ^58^ ^]^

### Golgi Staining

Golgi staining in brain tissue from 6‐month‐old mice was performed using a FD Rapid Golgi Stain Kit (FD Neuro Technologies, Columbia, MD). The cervical spine was quickly dislocated, and the brain was rapidly removed. Brains were embedded in A+B solution and incubated in the dark for 14 days. Subsequently, the brains were transferred to C solution and incubated in the dark for 5 days. Brain tissue was then cut into 100‐µm sections using a LEICA CM 1950 and stained with a D+E solution. Images were acquired as Z‐stacks using an Olympus IX81 inverted microscope (Olympus, Japan).

### TS Staining

Brain sections were incubated in 0.002% TS (Sigma‐Aldrich, catalog no. T1892‐25G) in 50% ethanol for 8 min. Co‐staining with other antibodies was carried out after three washes with PBS.

### Immunofluorescence

Immunofluorescence staining was performed as described before.^[^ ^15^ ^]^ Mice were perfused with saline, after which whole brains were freshly harvested and immediately fixed in 4% paraformaldehyde (*w*/*v*). Fixed tissue was dehydrated in 30% sucrose, subsequently cut into coronal sections, and stained using the antibodies listed in Table S2 (Supporting Information). Images were captured using a spinning disk confocal microscope (Andor, England, Oxford). Immunofluorescence images were analyzed using Image‐Pro 6.0, ImarisViewer 9.8.0, or Imaris 9.0.1 (Oxford Instruments).

### Real‐Time Quantitative RT‐PCR

Real‐Time Quantitative Reverse Transcription‐PCR (qRT‐PCR) was performed as previously described.^[^ ^15^ ^]^ Total RNA was extracted using TRIzol reagent (Invitrogen, catalog no. 15 596 018). First‐strand cDNA was synthesized from 1 µg of the extracted RNA using a cDNA synthesis kit from TIANGEN (catalog no. KR118). qRT‐PCR was performed on an Agilent Mx3005P RT‐PCR system. All the primers used are listed in Table S2 (Supporting Information).

### Extraction of Soluble and Insoluble Aβ

Brain samples were homogenized in 300 µL of RIPA buffer (25 mM Tris‐HCl, pH 7.5, 150 mM NaCl, 1% NP40, 0.5% NaDOC, 0.1% SDS), followed by centrifugation at 14 000 × *g* for 15 min. The supernatants were collected for the measurement of soluble Aβ. Insoluble Aβ was solubilized in 200 µL of SDS buffer (2% SDS, 25 mM Tris‐HCl, pH 7.5) and, after denaturation, the lysates were collected for the determination of insoluble Aβ using western blotting.

### Western Blotting

Western blotting was performed as previously detailed.^[^ ^15^ ^]^ Briefly, protein samples were boiled for 10 min at a temperature between 95 and 100 °C, separated by SDS‐PAGE, transferred to a membrane, and incubated with the primary antibody at 4 °C overnight. The next day, after washing three times, the membrane was incubated with horseradish peroxidase (HRP)‐conjugated secondary antibody at room temperature (RT) for 1 h. Finally, an enhanced chemiluminescent (ECL) substrate was added to the membrane, and the protein signal was detected using autoradiography film (Kodak, Rochester, NY).

### Microglia and Astrocyte Isolation from the Brains of Mice

Microglia and astrocytes were isolated from mouse brains using the Adult Brain Dissociation Kit (Miltenyi, 130‐107‐677) according to the manufacturer's protocol. Briefly, brain tissues from 6‐month‐old mice in each group were dissociated into single‐cell suspensions. Myelin and cell debris were removed using Debris Removal Solution, followed by erythrocyte elimination using Red Blood Cell Removal Solution. Microglia were isolated using mouse CD11b (Microglia) MicroBeads (Miltenyi Biotec, 130‐093‐634), and astrocytes were further purified using the Anti‐ACSA‐2 MicroBead Kit for mouse (Miltenyi Biotec, 130‐097‐678).

### RNA‐Seq and Bioinformatics Analysis

Microglia and astrocytes were isolated from three mice and pooled into one sample for RNA extraction and purification. RNA sequencing was performed by Majorbio Co. Ltd. (Shanghai, China), as previously described.^[^ ^59^ ^]^ Briefly, transcriptome libraries were prepared using 10 ng of total RNA and constructed according to Illumina's standard protocol. Paired‐end sequencing (2 × 150 bp) was carried out using the Illumina NovaSeq 6000 platform.

Differential gene expression analysis was performed using DESeq2, with an adjusted *P*‐value <0.05 and |log_2_FC| ≥0.5 as cutoffs. Pathway enrichment analysis was conducted using KOBAS 3.0. GO terms with a corrected *P*‐value <0.05 were considered significantly enriched. KEGG pathway analysis was performed based on the degree of enrichment of the differentially expressed genes. Heatmaps and volcano plots were generated using the Majorbio Bioinformatics Cloud Computing Platform.

### Primary Microglial Cell Preparation and Transfection

Primary microglia were prepared using neonatal (aged 1 to 3 days) *Rack1* WT mice and *Rack1* cKO mice. 4‐OH‐TAM was added to the primary microglia for 4 days of treatment to knock out *Rack1*. Equal amount of the primary microglial conditioned medium was then added to the primary astrocytes culture medium. After 24 h treatment, the changes in their proliferative and phagocytotic abilities were determined using an FITC‐Aβ phagocytosis assay and a CCK‐8 assay.

For siRNA transfection, primary microglia were seeded in a 12‐well plate and transfected with 50 nM siRNA using Lipofectamine RNAi MAX (Invitrogen, Waltham, MA, USA). After 72 h treatment, the knockdown deficiency was detected. The siRNA sequences are listed in Table S2 (Supporting Information).

### ELISA

The levels of IGF1 in primary microglia supernatant were detected using an IGF1 ELISA kit (Elabscience, E‐MSEL‐M0013).

### Phagocytosis Assay

Briefly, primary astrocytes were seeded in 24‐well plates and treated with primary microglial conditioned medium overnight. Then, 1 µg mL^−1^ of aggregated FITC‐Aβ_1–42_ (AnaSpec, AS‐60479) was added to the medium, followed by incubation in the dark for 3 h. After three PBS washes, astrocytes were fixed in 4% paraformaldehyde (*w*/*v*) at RT for 30 min, permeabilized with 0.2% Triton X‐100 (Sigma‐Aldrich, catalog no. V900502) in PBS at RT for 30 min, and incubated with the indicated antibody. Images were acquired using a spinning disk confocal microscope (Andor, England).

### CCK‐8 Assay

Briefly, 2 × 10^4^ astrocytes were seeded in 96‐well plates. Conditioned medium from primary microglia treated with siRNA for 72 h was then added to the astrocyte culture medium, followed by incubation for 24 h. Following incubation, CCK‐8 solution (10 µL) was added to each well, and the plates were incubated for 3 h. Absorbance was measured at 450 nm using a microplate reader (TECAN, Switzerland).

### Wound Healing Assay

The wound healing assay was performed as previously described.^[^ ^59^ ^]^ Briefly, a sterile 200‐µL pipette tip was used to create scratch wounds in cells cultured in 6‐well plates, and images were taken after 0, 6, 12, and 24 h using an optical microscope (Invitrogen EVOS, USA). Black lines were drawn to indicate the borders of the scratches, and the migration area was measured using Image‐Pro 6.0 software.

### Statistical Analysis

Data were analyzed using GraphPad Prism (GraphPad Software). Mann–Whitney U test was used for comparisons between two groups, while Kruskal–Wallis test was employed for comparisons among three or more groups. Two‐way ANOVA with Tukey's multiple comparisons test was used for two‐factors situations. Details of the tests used are provided in the figure legends. All values are presented as means ± SEM. A *P*‐value of <0.05 was considered significant.

## Conflict of Interest

The authors declare no conflict of interest.

## Author Contributions

J.Z. and Y.H. contributed equally to this work. J.Z. performed experiments, data analysis, and wrote the paper. Y.H., P.Y., H.Z., Y.T., L.J., Z.L., and M.Y. performed some experiments. X.L., J.Y., and Q.Y. analyzed data. J.Z. contributed new reagents. J.C. and Z.Y. designed research.

## Supporting information



Supporting Information

Supporting Information

Supporting Information

## Data Availability

The data that support the findings of this study are available from the corresponding author upon reasonable request.
