# Supplementary material for: Microglial Rack1 Deficiency Alleviates Alzheimer's Disease Pathology through Enhancing IGF1‐Mediated Astrocytic Phagocytosis
Source: Adv Sci (Weinh). 2025 Oct 30;13(3):e15877. doi: 10.1002/advs.202515877 (PMC12806348; doi:10.1002/advs.202515877)
Supplement: Supplementary file 1 — Supporting Information [file ADVS-13-e15877-s001.docx]

**Supplementary Figure legends**

**Figure S1. The expressions of Rack1 in microglia in WT and AD mouse.** (**A, B**) Increase of mRNA levels of *Rack1* in isolated microglia in WT (N = 2 from 6 mice) and AD mouse (N = 2 from 6 mice). (**C，D**) The cell types in *5×FAD* and control brain samples. (**E, F**) The Rack1 levels in different cell type and different microglial subclusters (Data form Hadas Keren-Shaul et al, GEO: GSE98969). The mouse Single-cell sequencing datasets and analyzed by package Seurat (v4.1.0). Quality control and each cell cluster were based on the original articles. All values are presented as mean ± SEM. **P* < 0.05 and ****P* < 0.001. Descriptive statistics (B), and Wilcox test (F).

**Figure S2.** **The** **knockout efficiency of Rack1.** (**A**) Immunofluorescent staining of Rack1 and Iba1, and statistical analysis of the co-expression of Rack1 and Iba1 in the hippocampus of 6-month-old *Rack1* WT mice and *Rack1* cKO mice (n = 6 mice per group). (**B**) Rack1-positive microglia in Iba1-positive microglia. (**C**) Rack1-positive astrocyte in GFAP-positive astrocyte. (**D**) qPCR assay to test the expression levels of *Rack1* in isolated microglia from these four groups of mice (n = 3 per group). All values are presented as mean ± SEM. ***P* < 0.01 and ****P* < 0.001. Mann-Whitney U test (B and C), and Kruskal-Wallis test (D).

**Figure S3.** **Interaction time in NOR test of four group mice.** (A) The interaction time in NOR test of 6-month-old *Rack1* WT mice (n = 7), *Rack1* cKO mice (n = 8), *Rack1* WT/AD mice (n = 7), and *Rack1* cKO/AD mice (n = 10). All values are presented as mean ± SEM. Kruskal-Wallis test (A).

**Figure S4.** **Conditional knockout of microglial *Rack1* does not affect APP metabolism.** (**A** to **D**) Immunoblotting and statistical analysis of levels of Nicastrin, APP, and PSEN2 in the hippocampus of 6-month-old *Rack1* WT, *Rack1* cKO, *Rack1* WT/AD, and *Rack1* cKO/AD. The protein levels were quantified by software Image J. Each point in the figure represents the relative protein level from one brain. All values are presented as mean ± SEM. ***P* < 0.01 and ****P* < 0.001. Kruskal-Wallis test (B-D).

**Figure S5.** **Conditional knockout of microglial *Rack1*** **decreases microglia number, phagocytosis and proliferation.** (**A, B**) Immunofluorescent staining and statistical analysis of Iba1-positive cells in DG, CA1 and PFC of these four 6-month-old groups mice as indicated (n = 6 mice per group). (**C, D**) TS staining, immunofluorescent staining of Iba1 and TREM2, and statistical analysis of TREM2 density in microglia around Aβ plaque in PFC of 6-month-old *Rack1* WT/AD mice and *Rack1* cKO/AD mice (n = 6 mice per group). (**E, F**) TS staining, immunofluorescent staining of Iba1 and CD68, and statistical analysis of CD68 density in microglia around Aβ plaque in PFC of 6-month-old *Rack1* WT/AD mice and *Rack1* cKO/AD mice (n = 6 mice per group). All values are presented as mean ± SEM. **P* < 0.05, ***P* < 0.01, and ****P* < 0.001. Two-way ANOVA with Tukey’s multiple comparisons test (B), and Mann-Whitney U test (D and F).

**Figure S6.** **Knockdown of Rack1 in BV2 cell reduces cell migration, and decreases inflammation.** (**A**) qPCR assay to test the knockdown efficiency of Rack1. (**B**) The effect of knockdown Rack1 in microglia using CCK8 assay as indicated. (**C, D**) A scratch was made in the middle of the wells with wound maker tool. Cell confluency within the scratch area was analyzed at indicated times. (**E**) Schematic for LPS treatment in control and Rack1 knockdown BV2 cells. (**F** to **H**) qPCR assay to test the expression levels of IL-1β, INOS, TNF-α in control and Rack1 knockdown BV2 cell lines. All values are presented as mean ± SEM. **P* < 0.05, ***P* < 0.01, and ****P* < 0.001. Mann-Whitney U test (A), and Two-way ANOVA with Tukey’s multiple comparisons test (B, D, F-H).

**Figure S7.** **Conditional knockout of microglial *Rack1*** **increases astrocytic proliferation.** (**A, B**) TS staining, immunofluorescent staining of GFAP, and Ki67 staining in 6-month-old *Rack1* WT/AD mice and *Rack1* cKO/AD mice and statistical analysis of Ki67 density in GFAP-positive cells (n = 6 mice per group). All values are presented as mean ± SEM. **P* < 0.05 and ****P* < 0.001. Mann-Whitney U test (B).

**Figure S8.** **Volcano plot and KEGG analysis of RNA sequencing data and conditional knockout of microglial *Rack1*** **increases astrocyte autophagy.** (**A, B**) Volcano plot of microglia and astrocyte RNA sequencing data shows two groups of expressed genes between *Rack1* WT/AD mice and *Rack1* cKO/AD mice (log_2_ fold change ≥ 0.5, adjusted false discovery rate < 0.05). (**C**) KEGG enrichment of upregulated gene analysis reveals upregulated signaling pathways in microglia in 6-month-old *Rack1* cKO/AD mice compared with *Rack1* WT/AD mice (log_2_ fold change ≥ 0.5, adjusted false discovery rate < 0.05). (**D**) KEGG enrichment of upregulated gene analysis reveals upregulated signaling pathways in astrocyte in 6-month-old *Rack1* cKO/AD mice compared with *Rack1* WT/AD mice (log_2_ fold change ≥ 0.5, adjusted false discovery rate < 0.05). (**E**) TS staining, immunofluorescent staining of GFAP and LC3 within the PFC of 6-month-old *Rack1* WT/AD mice and *Rack1* cKO/AD mice. (**F**) Aβ staining, immunofluorescent staining of GFAP and p-Atg16L1 within the PFC of 6-month-old *Rack1* WT/AD mice and *Rack1* cKO/AD mice. (**G, H**) Statistical analysis of LC3-positive, and p-Atg16L1-positive around Aβ plaque (n = 6 mice per group). All values are presented as mean ± SEM. **P* < 0.05 and ****P* < 0.001. Mann-Whitney U test (G, H).

**Figure S9.** **Heatmap and qPCR assay of RNA sequencing data.** (**A, B**) Heatmap and qPCR assay to test the expression levels of Ccl3, Ccl4, IL1β, and Nos2 in microglia from 6-month-old *Rack1* WT/AD mice and *Rack1* cKO/AD mice. All values are presented as mean ± SEM. ***P* < 0.01 and ****P* < 0.001. Mann-Whitney U test (B).

**Figure S10.** **Inhibition of IGF1R partially alleviates the autophagy of astrocyte in microglial *Rack1* deficiency *5×FAD* mice.** (**A, B**) TS staining, Immunofluorescence staining of LC3-positive cells within the PFC of 5-month-old *Rack1* WT/AD mice, *Rack1* cKO/AD mice, *Rack1* WT/AD+PPP mice, and *Rack1* cKO/AD+PPP mice and statistical analysis of LC3-positive in astrocyte around Aβ plaque (n = 3 mice per group). All values are presented as mean ± SEM. **P* < 0.05 and ***P* < 0.01. Kruskal-Wallis test (B).

**Figure S11.** **Inhibition of IGF1R partially blocks the response of microglia.** (**A**, **B**) Immunofluorescent staining and statistical analysis of Iba1-positive cells in DG, CA1 and PFC of these four group mice as indicated (n = 3 mice per group). (**C**, **D**) TS staining, immunofluorescent staining of Iba1, and statistical analysis of microglia number per Aβ plaque in PFC as indicated (n = 3 mice per group). (**E**, **F**) TS staining, immunofluorescent staining of Iba1 and TREM2, and statistical analysis of TREM2 density in microglia around Aβ plaque in PFC as indicated (n = 3 mice per group). (**G**, **H**) TS staining, immunofluorescent staining of Iba1 and CD68, and statistical analysis of CD68 density in microglia around Aβ plaque in PFC as indicated (n = 3 mice per group). All values are presented as mean ± SEM. **P* < 0.05, ***P* < 0.01, and ****P* < 0.001. Two-way ANOVA with Tukey’s multiple comparisons test (B), and Kruskal-Wallis test (D, F and H).

**Figure S12.** **IGF1R inhibition negates the cognitive benefits of RACK1 deficiency in 5×FAD mice.** (**A**) MWM analysis of latency (s) to target in the platform training. (B to D) MWM analysis of the latency (s), target cross number, and mean speed (cm/s) in the platform tests in 5-month-old *Rack1* WT/AD mice (n=7), *Rack1* cKO/AD mice (n=8), *Rack1* WT/AD+PPP mice (n=7), and *Rack1* cKO/AD+PPP mice (n=7). (E) NOR analysis of the recognition index in 5-month-old *Rack1* WT/AD mice (n=7), *Rack1* cKO/AD mice (n=8), *Rack1* WT/AD+PPP mice (n=7), and *Rack1* cKO/AD+PPP mice (n=7). All values are presented as mean ± SEM. *P < 0.05, and **P < 0.01. Two-way ANOVA with Tukey’s multiple comparisons test (A), and Kruskal-Wallis test (B-E).
