# Supplementary figures and images for: Microglial Rack1 Deficiency Alleviates Alzheimer's Disease Pathology through Enhancing IGF1‐Mediated Astrocytic Phagocytosis
Source: Adv Sci (Weinh). 2025 Oct 30;13(3):e15877. doi: 10.1002/advs.202515877 (PMC12806348; doi:10.1002/advs.202515877)

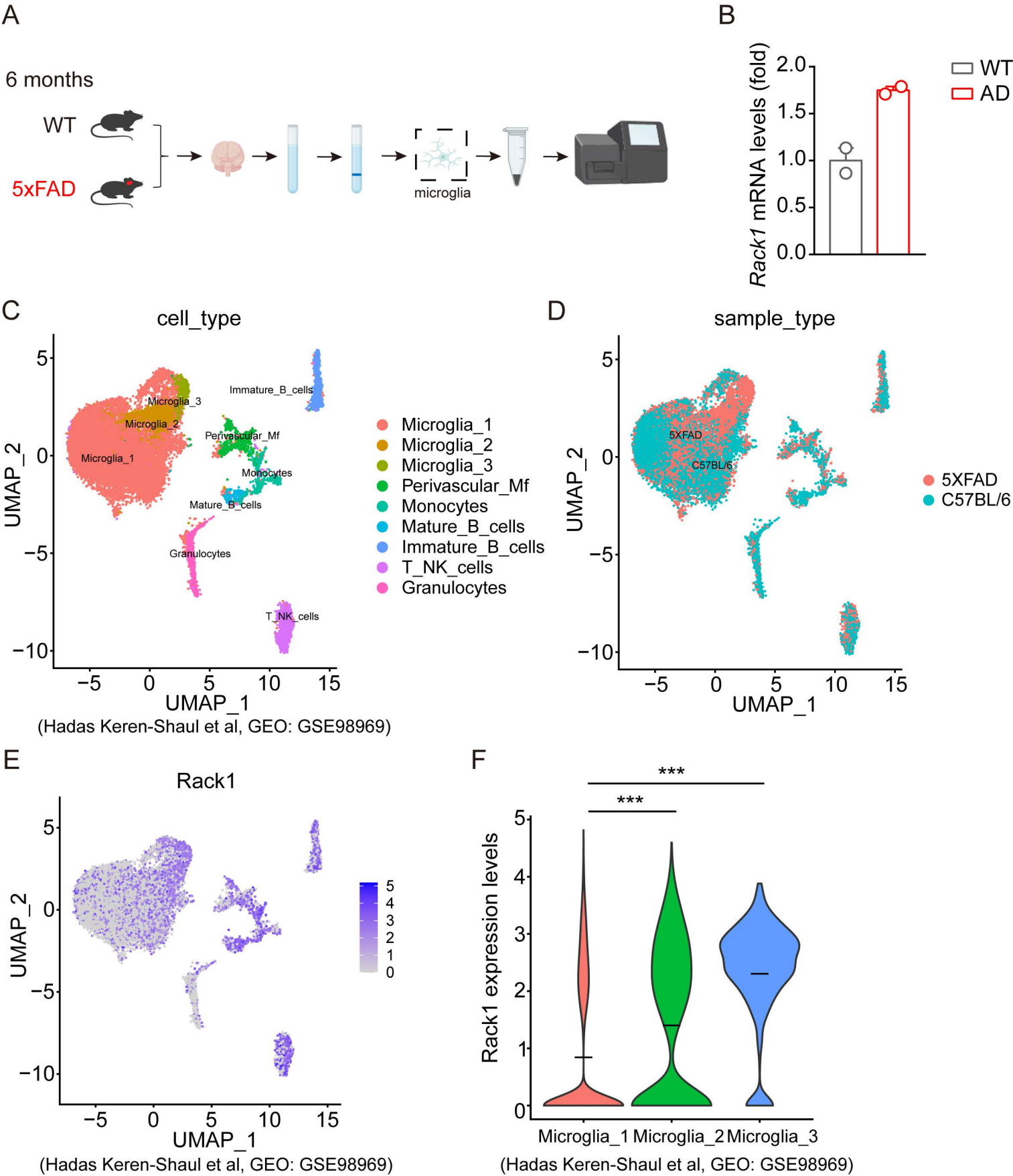

A

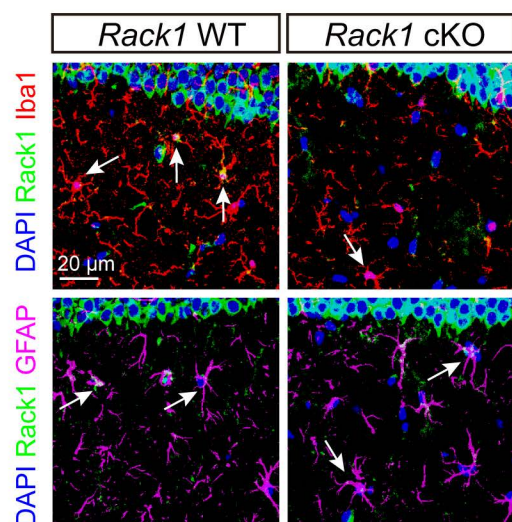

B

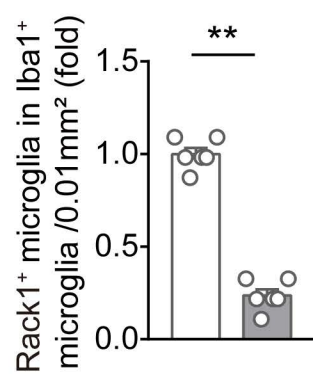

C

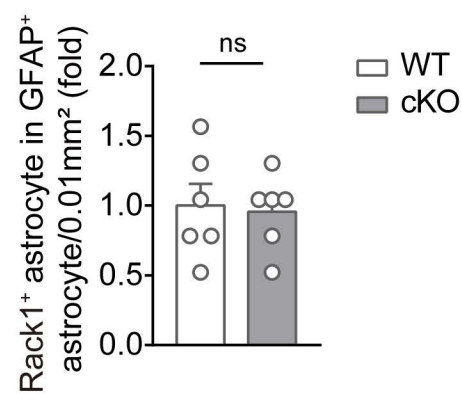

D

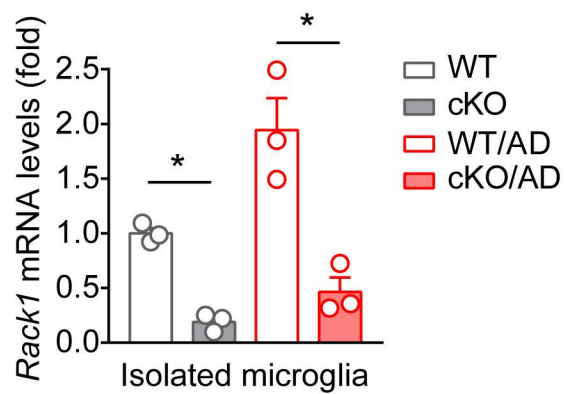

A

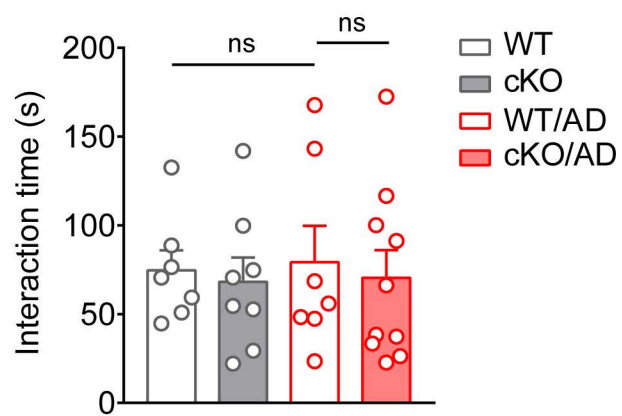

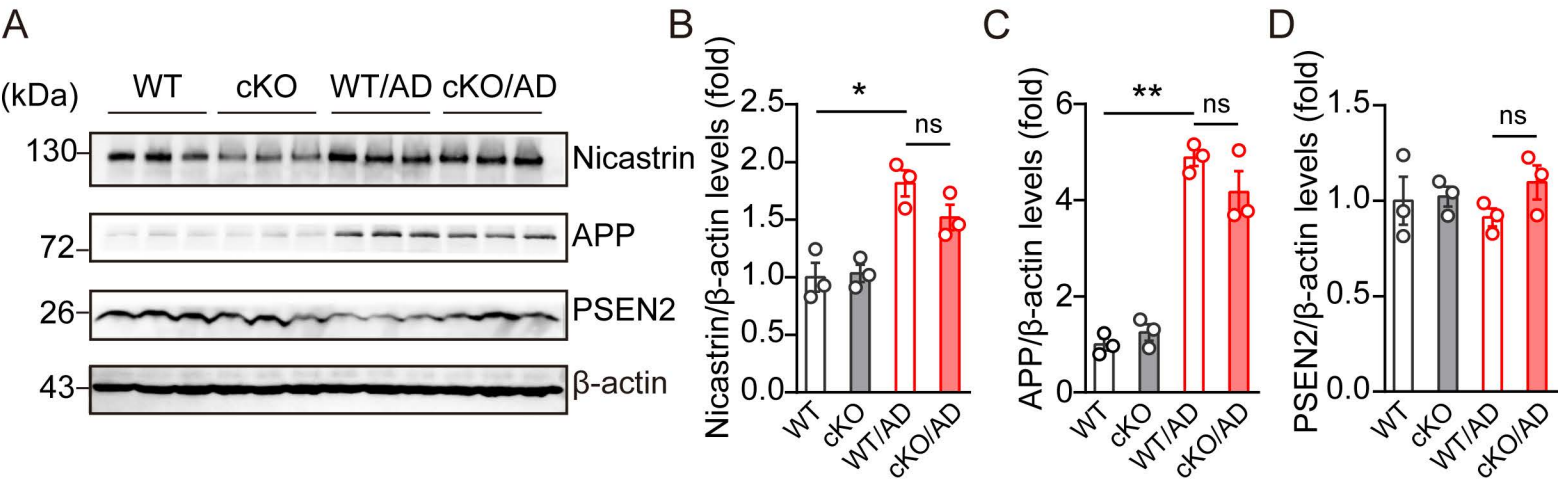

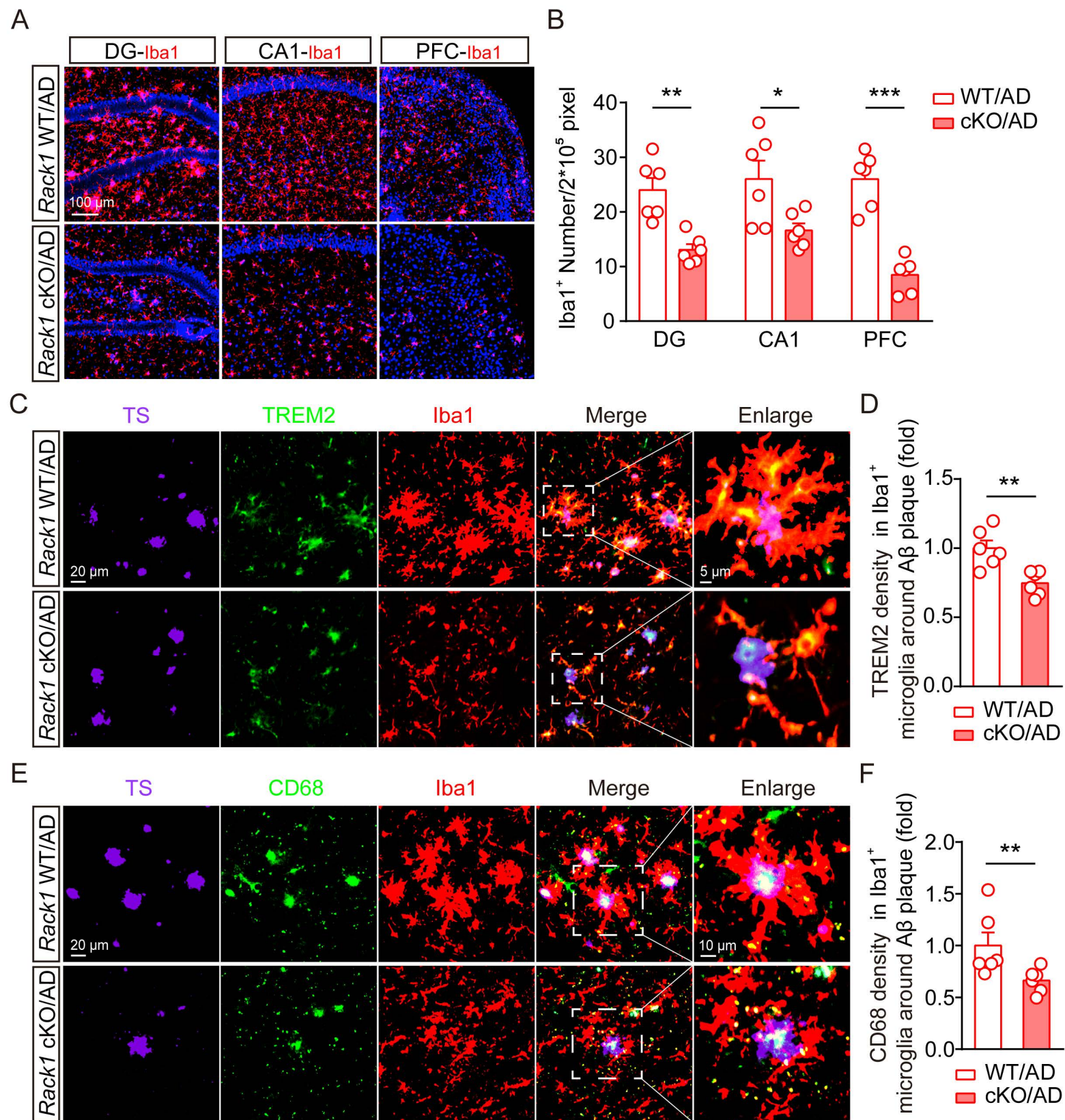

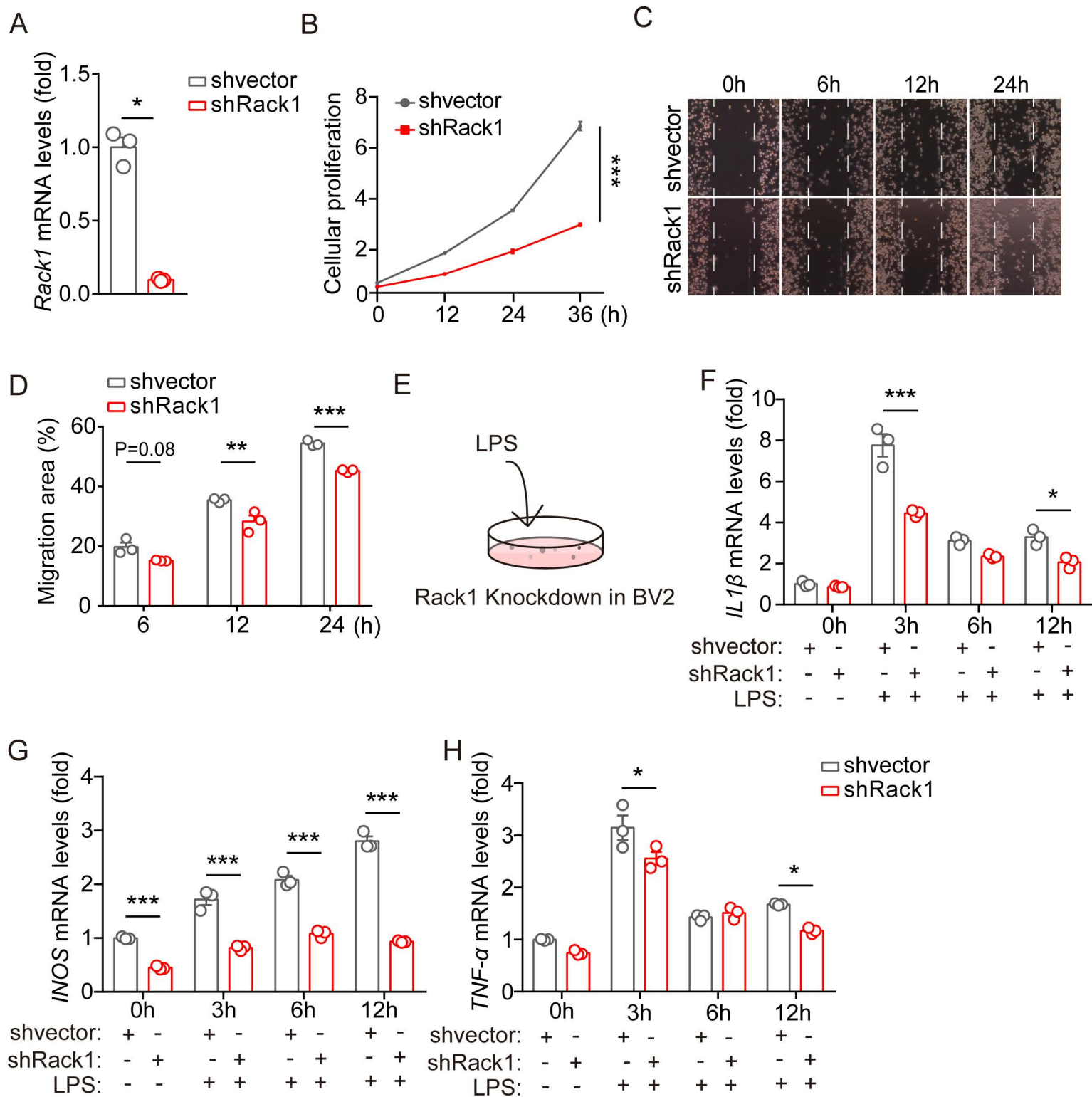

A

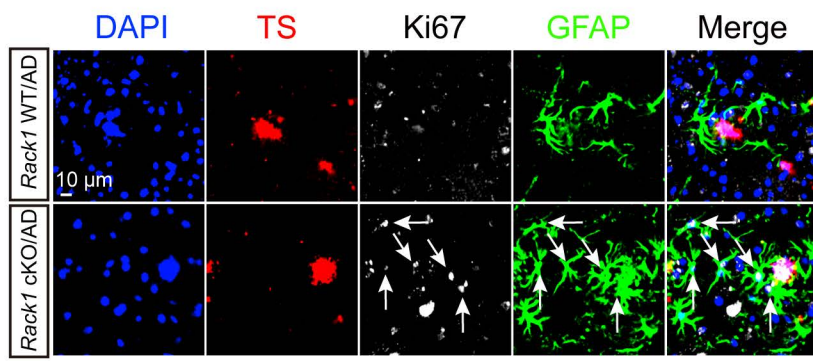

B

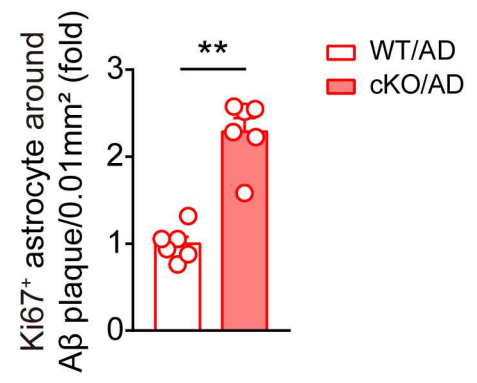

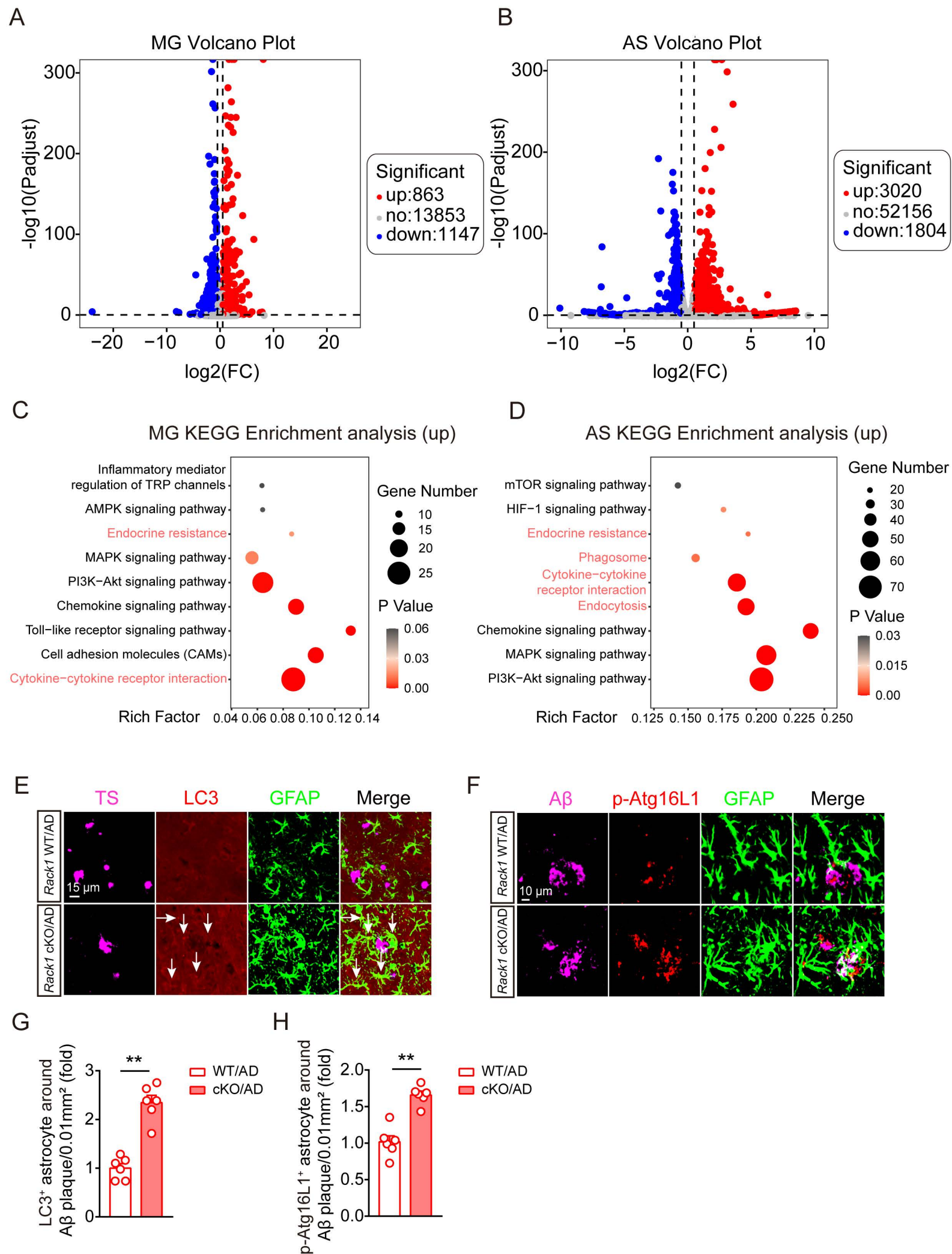

A

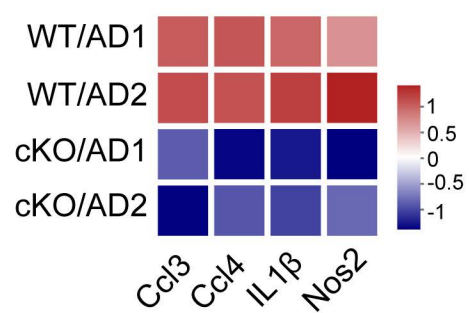

B

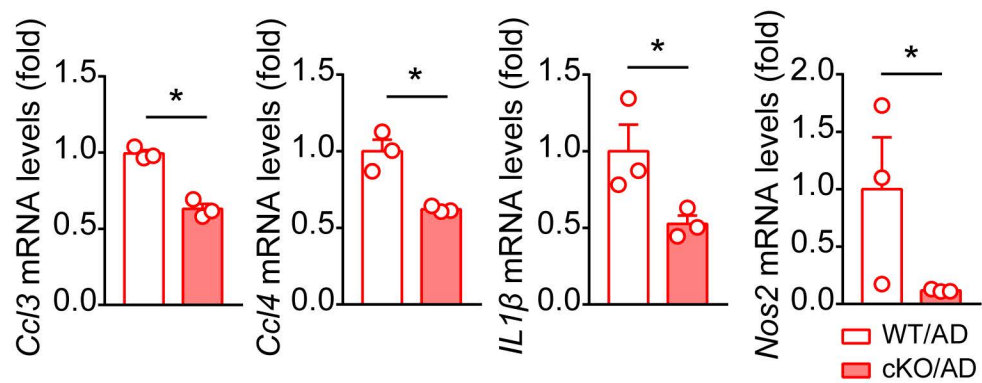

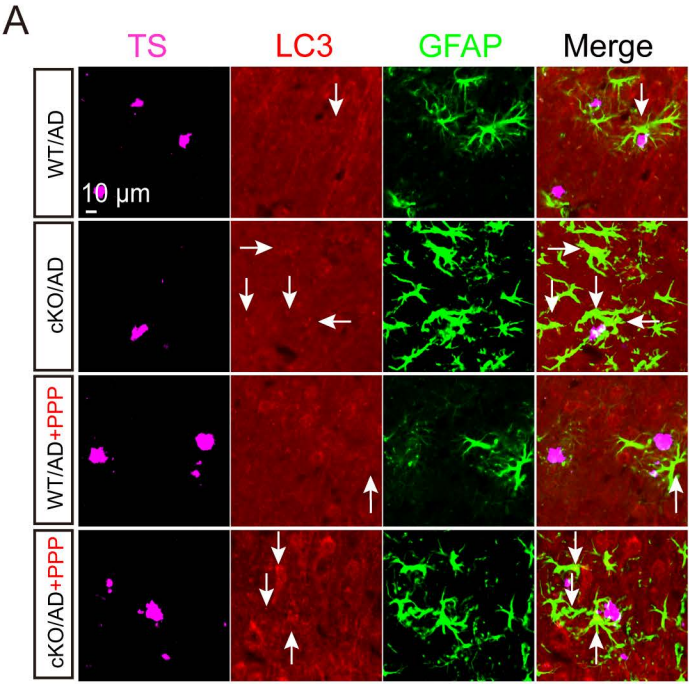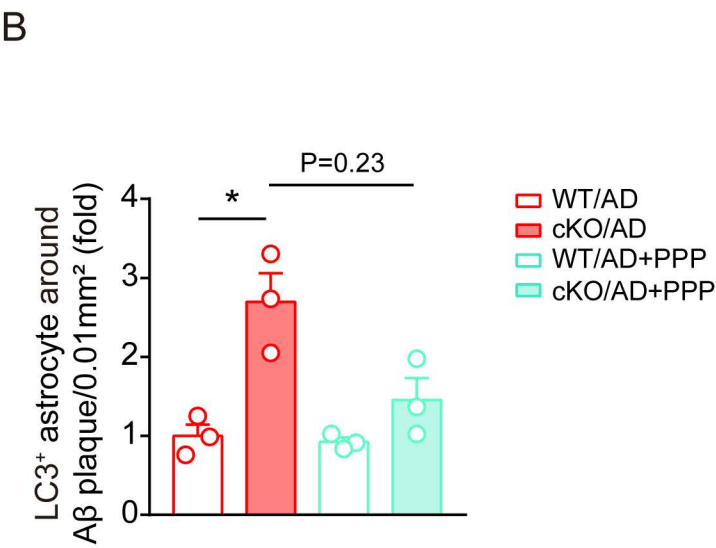

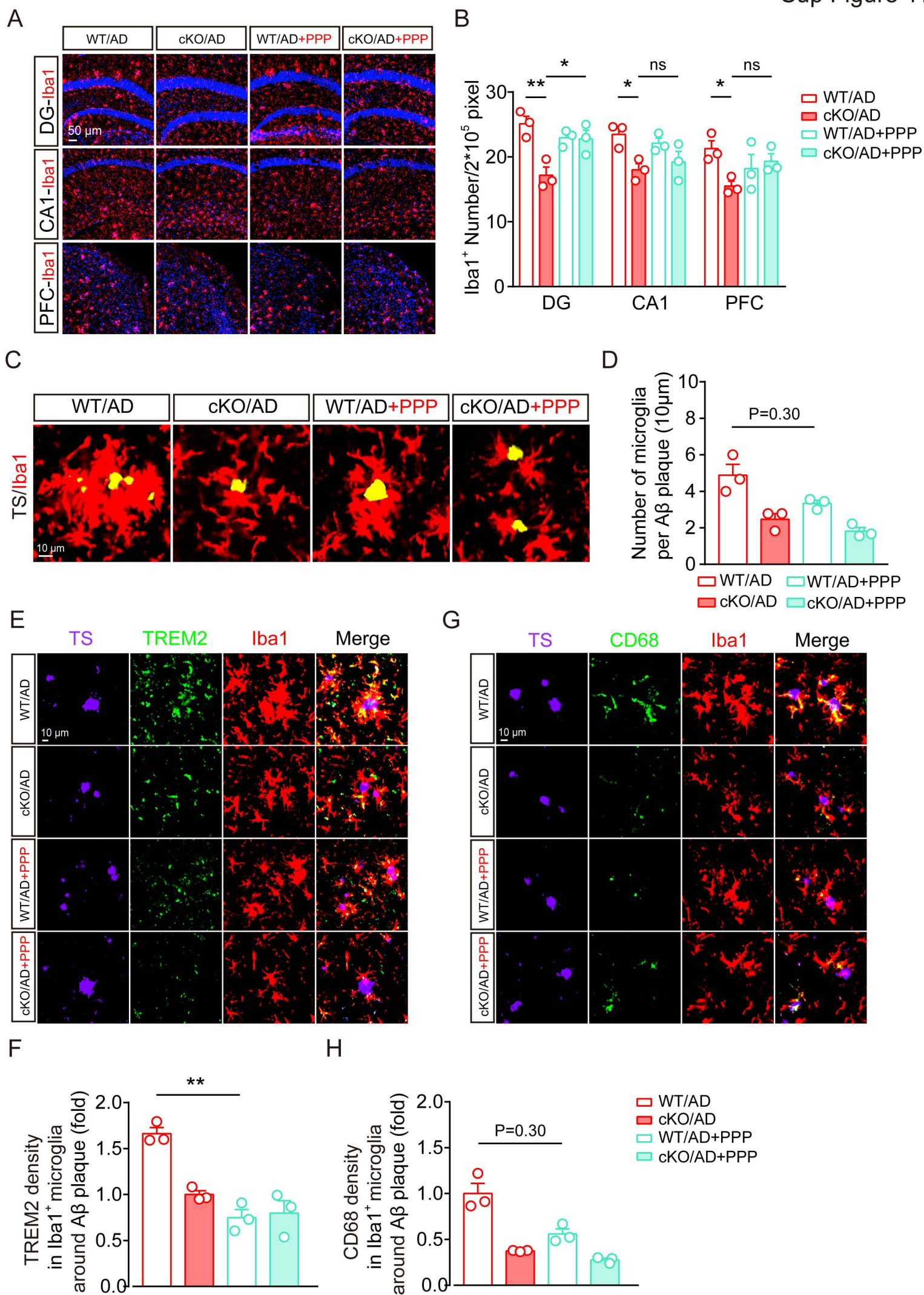

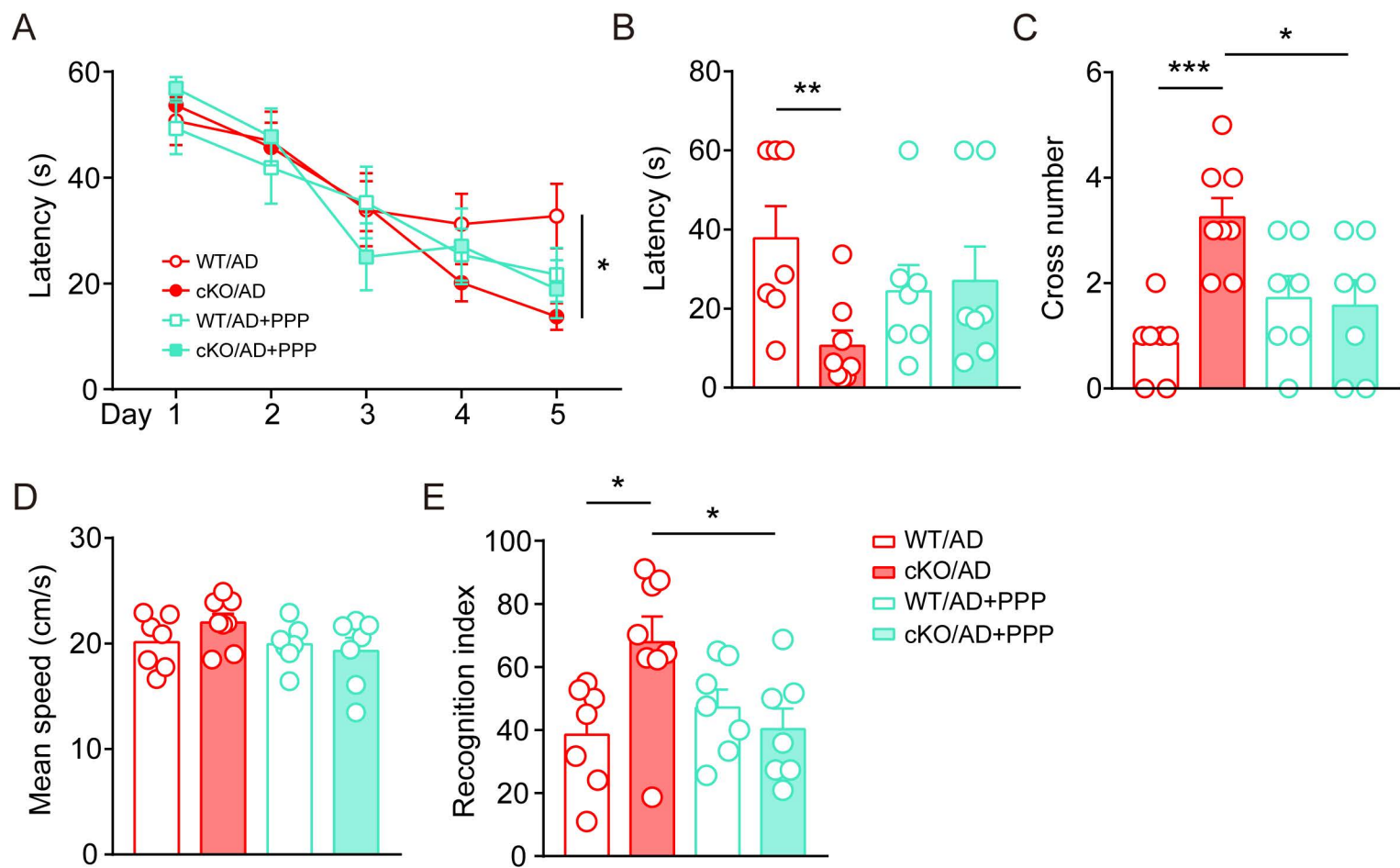

Supplement: Supplementary file 2 — Supporting Information [file ADVS-13-e15877-s002.pdf]
