# Supplementary material for: Microglial Rack1 Deficiency Alleviates Alzheimer's Disease Pathology through Enhancing IGF1‐Mediated Astrocytic Phagocytosis
Source: Adv Sci (Weinh). 2025 Oct 30;13(3):e15877. doi: 10.1002/advs.202515877 (PMC12806348; doi:10.1002/advs.202515877)
Supplement: Supplementary file 3 — Supporting Information [file ADVS-13-e15877-s003.docx]

**Suppl. Table 1:** The information of brain samples of control and patients with AD.

| Type | Gender | Age | Tissue |
| --- | --- | --- | --- |
| Control | Male | 78 | Hippocampus |
| Control | Male | 93 | Hippocampus |
| Control | Male | 95 | Hippocampus |
| Control | Male | 89 | Hippocampus |
| Control | Male | 70 | Hippocampus |
| AD patient | Male | 93 | Hippocampus |
| AD patient | Male | 94 | Hippocampus |
| AD patient | Male | 87 | Hippocampus |
| AD patient | Male | 77 | Hippocampus |
| AD patient | Male | 78 | Hippocampus |

**Suppl. Table 2:** The information of reagents or resource.

| Reagents or resource | Source | Identifier |
| --- | --- | --- |
| Antibody | | |
| Rack1 | BD Biosciences | 610177 |
| GFAP | Dako | Z0334 |
| Iba1 | Novus | NB100-1028 |
| TREM2 | R&D | AF1729 |
| CD68 | Abcam | ab53444 |
| 6E10 (Aβ &sAPPα) | Covance | SIG-39320 |
| CTSD | R&D | AF1029 |
| LC3 | Proteintech | 14600-1-AP |
| p-Atg16L1 | Abcam | Ab195242 |
| Ki67 | Cell Signaling Technology | 12202S |
| β-actin | Proteintech | 66009-1-lg |
| APP | Cell Signaling Technology | 2452 |
| Nicastrin | Cell Signaling Technology | 5887 |
| PSEN2 | Cell Signaling Technology | 5887 |
| Alexa Fluor 488 donket anti-mouse IgG (H+L) | Invitrogen | A21202 |
| Alexa Fluor 568 donket anti-rabbit IgG (H+L) | Invitrogen | A10042 |
| Alexa Fluor 647 donket anti-goat IgG (H+L) | Invitrogen | A21447 |
| Chemicals & probes | | |
| TS | Sigma-Aldrich | T1892-25G |
| DAPI | Yeason | WD8310030 |
| Tamoxifen | Sigma-Aldrich | T5648 |
| 4-Hydroxytamoxifen | Selleck | S782702 |
| LPS | Merck Millipore | LPS25 |
| FITC-Aβ 1-42 | AnaSpec | AS-60479 |
| Triton X-100 | Sigma-Aldrich | V900502 |
| Picropodophyllin | Selleck | S7668 |
| CD11b MicroBeads | Miltenyi Biotec | 130-093-634 |
| Anti-ACSA-2 MicroBeads | Miltenyi Biotec | 130-097-678 |
| ELISA kit | | |
| IGF1 | Elabscience | E-MSEL-M0013 |
| Primers | | |
| Mouse *Rack1* | Forward | 5’-AGGGCCACAATGG  ATGGGTA-3’ |
|  | Reverse | 5’-TCTGGTCAGCTTC  CACATGAT-3’ |
| Mouse *β-actin* | Forward | 5’-GGCTGTATTCCCCT  CCATCG-3’ |
|  | Reverse | 5’-CCAGTTGGTAACA  ATGCCATGT-3’ |
| Mouse *GAPDH* | Forward | 5’-AGGTCGGTGTGAA  CGGATTTG-3’ |
|  | Reverse | 5’-GGGGTCGTTGATG  GCAACA-3’ |
| Mouse *IGF1* | Forward | 5’-CTGGACCAGAGAC  CCTTTGC-3’ |
|  | Reverse | 5’-GGACGGGGACTTC  TGAGTCTT-3’ |
| Mouse *IGF1r* | Forward | 5’-GTGGGGGCTCGTG  TTTCTC-3’ |
|  | Reverse | 5’-GATCACCGTGCAG  TTTTCCA-3’ |
| Mouse *Nos2/INOS* | Forward | 5’-ATGGACCAGTATA  AGGCAAGC-3’ |
|  | Reverse | 5’-GCTCTGGATGAGC  CTATATTG-3’ |
| Mouse *IL-1β* | Forward | 5’-TGTAATGAAAGAC  GGCACACC-3’ |
|  | Reverse | 5’-TCTTCTTTGGGTAT  TGCTTGG-3’ |
| Mouse *TNF-α* | Forward | 5’-CAGGCGGTGCCTA  TGTCTC-3’ |
|  | Reverse | 5’-CGATCACCCCGAA  GTTCAGTAG-3’ |
| Mouse *Ccl3* | Forward | 5’-TTCTCTGTACCAT  GACACTCTGC-3’ |
|  | Reverse | 5’-CGTGGAATCTTCC  GGCTGTAG-3’ |
| Mouse *Ccl4* | Forward | 5’-TTCCTGCTGTTTCT  CTTACACCT-3’ |
|  | Reverse | 5’-CTGTCTGCCTCTTT  TGGTCAG-3’ |
| Mouse siRack1 | siRack1 | 5’-GUAGAUGAAUUG  AAGCAAG-3’ |
